# Seedy Banana – A Source of Stilbenes and Flavan-3-ols

**DOI:** 10.1021/acs.jafc.5c01416

**Published:** 2025-06-06

**Authors:** Hoai Thi Tran, Markus Bacher, Stefano Barbini, Thomas Rosenau, Stefan Böhmdorfer

**Affiliations:** † Institute of Chemistry of Renewable Resources, BOKU University, Konrad-Lorenz-Straße 24, Tulln 3430, Austria; ‡ Faculty of Food Science and Technology, Vietnam National University of Agriculture, Hanoi 100 000, Vietnam; § NATEX Prozesstechnologie GesmbH, Werkstrasse 7, Ternitz 2630, Austria

**Keywords:** piceatannol, epiafzelechin, seedy banana, phenolic compounds, atropisomerism, biorefinery, bioactive compounds

## Abstract

This study aimed at identifying the bioactive compounds in the seeds of three seedy banana varietiesMusa acuminata, Musa itinerans, and Ensete glaucum. GC-MS of extracts and NMR of isolated compounds were used for the identification of the components and GC-MS/FID was employed for quantification. The proportion of seeds in all samples was around 50% of the dried fruit weight. The seeds were rich in piceatannol, (+)-epiafzelechin, and epiafzelechin dimers. Piceatannol and epiafzelechin dimers were reported in seedy bananas for the first time. The highest piceatannol content was found in Ensete, accounting for 713 ± 71.9 mg/100 g dry weight (DW), followed by the seeds of ripe Musa acuminata and Musa itinerans at 440 ± 56.7 and 242 ± 17.4 mg/100 g DW, respectively. Musa seeds with a higher content of piceatannol had lower concentrations of epiafzelechin and its dimers, in the ranges of 287 to 553 mg/100 g of DW and 533 to 976 mg/100 g of DW, respectively. The content depended on maturity. Compared with ripe fruits, green Musas contained hardly any piceatannol and higher levels of epiafzelechin dimers. Gallocatechin was detected only in Ensete glaucum at 145 ± 19.7 mg/100 g of DW, and a major part of the phenolic compounds in g was found in the seed coat. The identified phenolic compounds in the seeds suggest that seedy bananas could be a potential new crop for food supplements.

## Graphical abstract

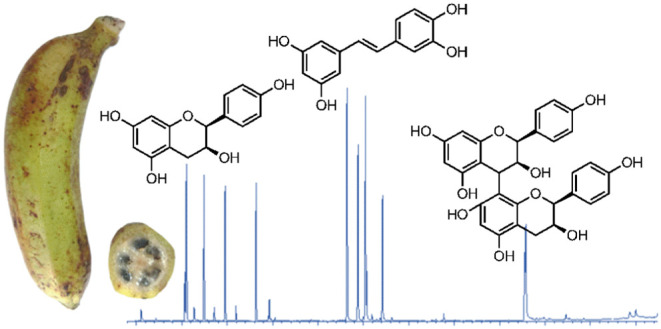

## 1 Introduction

Seedy banana is a wild-growing diploid plant that originated in Southeast Asia and the surrounding tropical and subtropical areas. It belongs to the order Zingiberales and the family Musaceae.^1,2^ This family includes three genera: Musa, Ensete, and Musella, with Musa being the largest group. Seedy banana has been in use for a long time as a traditional medicine in different countries.^3,4^ In India, seedy bananas are used against pinworm infection, cough, dysentery, stomach disorders, and respiratory tract disorders.^3^ In Vietnam, ripe fruits are eaten in the same way as dessert bananas and have been traditionally used to treat diabetes and kidney stones.^4^ However, the use of this plant in folk medicine still lacks scientific proof and is not based on knowledge of pharmaceutically active compounds. The seeds of Musa balbisiana have been shown to contain esters of phytol, sterol, and epiafzelechin.^5,6^ For comparison, studies on the triploid dessert banana fruits have described them as being rich in phenolic compounds, such as gallocatechin, epicatechin, catechin, proanthocyanin, gallic acid, ferulic acid, synaptic acid, and rutin.^7–9^ This suggests that bioactive compounds should also be found in seedy bananas by a thorough investigation.

Phenolic compounds, such as stilbenes or flavan-3-ols, represent the largest group of plant secondary metabolites.^10^ Stilbenes are produced naturally by plants and can be found both in free forms, such as resveratrol and piceatannol, as well as in glycosylated forms.^11^ Flavan-3-ols are commonly present in the monomeric form (e.g., catechin, epiafzelechin) and can also be found in polymeric forms (proanthocyanidins).^12^ Phenolic compounds generally have antioxidant properties, since the hydroxyl group of the phenolic can react with a free radical to form a radical species, in which the unpaired electron is delocalized over the aromatic ring. Also, some phenolics can reduce harmful oxidants and form quinones in the process. Studies on plant-derived polyphenols consistently demonstrate that phenolic compounds offer protection against diabetes and offer benefits against inflammation, cancer, and cardiovascular diseases.^13–15^ The investigation of plants for phenolics can, therefore, indicate the presence of health-promoting plant constituents.

Among the analytical methods that are used to determine chemical compounds in medicinal plants, gas chromatography hyphenated to mass spectrometry (GC-MS) is a method of choice due to its high separation efficiency and suitability for a wide range of compounds combined with direct compound identification. GC-MS mass spectral libraries have been assembled to serve as references for the tentative structure assignment of known constituents.^16^ This initial exploration can be followed by the confirmation of the structure of purified compounds by nuclear magnetic resonance (NMR) spectroscopy.

This study addresses the bioactive compounds in seedy bananas and their content. The distribution across the banana fruit and possible differences among varieties of seedy bananas were related research questions.

## 2 Material and Methods

### 2.1 Sample Collection and Preparation

Fruits of three seedy banana species, Musa itinerans, Musa acuminata, and Ensete glaucum, were collected in the wild at the Centre Highland region of Daklak province in December 2021 in Vietnam. The plants were identified by morphological comparisons of leaves, flowers, fruits, and seeds with the description of Hastuti et al.,^17^ Vu et al.,^18^ and Majumdar et al.,^19^ respectively (Figure 1). The fruits were collected at the green and ripe maturity stages: 6 bunches of Musa acuminata (3 ripe, 3 green), 4 bunches of Musa itinerans (1 ripe, 3 green), and 1 green bunch of Ensete glaucum. Of each bunch, the first two hands were taken and placed into separate paper bags that allowed good aeration. The samples were transported to the laboratory in Vietnam the following day. From each sampled bunch, 3–6 fruits were weighed and freeze-dried. The dried samples were transported to the laboratory in Austria. Pulp, peel, and seeds were then separated and weighed individually. The samples were ground separately using a Retsch Ultra Centrifugal Mill ZM 200 (Germany, 0.5 mm sieve) and stored at −80 °C before analysis.

**Figure 1:**
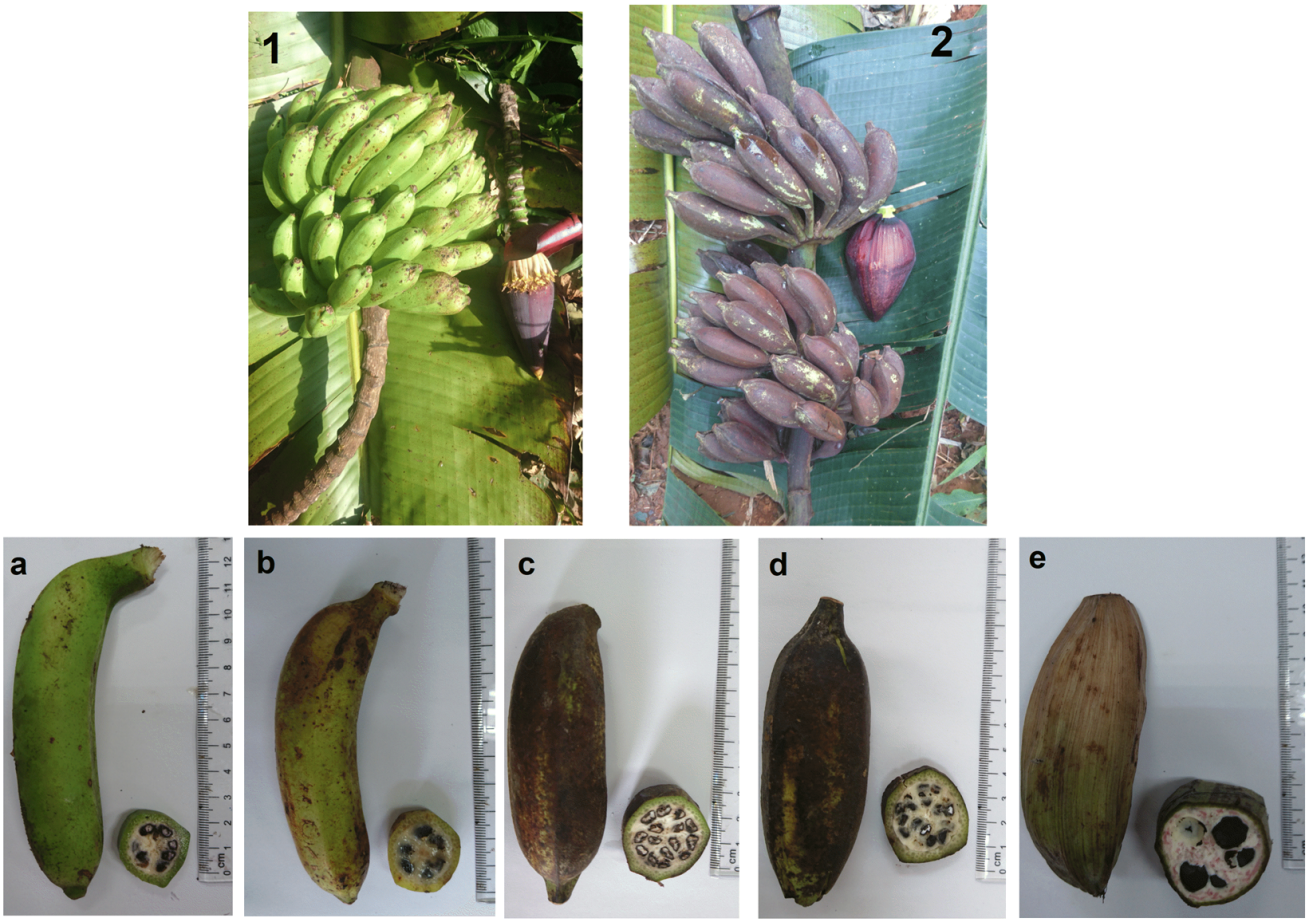
Bunches of Musa acuminata (1) and Musa itinerans (2). Fruits of Musa acuminata at green (a) and ripe (b) maturity stages, fruits of Musa itinerans at green (c) and ripe (d) maturity stages, and fruits of green Ensete glaucum (e).

### 2.2 Chemicals and Reagents

Acetone and formic acid were purchased from Sigma-Aldrich (Germany). Methanol and chloroform were purchased from Fisher (Germany). DMSO-*d*_6_, acetone-*d_6_* and methanol-*d*_4_ were obtained from Eurisotop (France). The derivatization reagents *N*,*O*-bis­(trimethylsilyl)­trifluoroacetamide (BSTFA, ≥99%), trimethylchlorosilane (TMCS, ≥99%), and pyridine were bought from Sigma-Aldrich (Germany). Standards including d-glucose, d-fructose, and *trans*-resveratrol were purchased from Sigma-Aldrich. d-Saccharose was purchased from Carl Roth (Germany).

### 2.3 Accelerated Solvent Extraction (ASE) of Seeds

Extraction was done on a Dionex ASE 350 (Thermo Scientific, Sunnyvale, CA) instrument with two different solvents in sequence: acetone followed by 70% aqueous methanol. An initial defatting step with hexane was not required, since the extraction with hexane yielded only 0.13% of oil. Approximately 4 g of seed powder was weighed into the extraction cell, and the ASE conditions were set to 50 °C, with a heating time of 5 min and a static time of 7 min. For each solvent, the sample was extracted 3 times. The supernatants from the three successive solvent extractions were combined into a single extract. In total, 160 mL of acetone extract and 160 mL of methanol extract were obtained. The extracts were evaporated on a rotary evaporator (Büchi, Switzerland) at 40 °C *in vacuo* (acetone: 55.6 kPa (556 mbar); aqueous methanol: 33.7 to 7.4 kPa (337 to 74 mbar)).

### 2.4 Identification and Quantification of Main Compounds in the Extract

#### 2.4.1 Derivatization

Derivatization was performed as described by Barbini et al.^20^ with minor modifications. In a 1.5 mL GC vial, the extract (1.5–2.5 mg) was mixed with pyridine (150 μL) and a derivatizing agent (9:1 (v/v) of BSTFA and TMCS, 200 μL). Resveratrol (0.1 mg) was added as an internal standard. The second internal standard that is part of the employed protocol, margaric acid, was not used for the quantification of the phenolics. The vials were vortexed and heated to 70 °C for 3 h.

#### 2.4.2 GC-MS/FID analysis

The derivatized samples were injected into a GC-MS/FID Agilent 7890A system (Agilent Technologies, Santa Clara, CA) equipped with an MSD detector (Agilent 5975C). Injection (1 μL) was performed using an autosampler into a cold multimode inlet (MMI). The inlet was kept at 65 °C for 6 s and then heated to 380 °C at 500 °C/min. This temperature was held for 5 min. The split ratio was set at 15:1 (split flow: 37.5 mL/min). A DB-5HT capillary column (30 m × 250 μm internal diameter, 0.1 μm film thickness) (Agilent Technologies, Santa Clara, CA) was used for separation. The system used helium as the carrier gas with a constant column flow of 2.5 mL/min. The oven temperature program was as follows: the initial temperature was 65 °C for 5 min, increased at 10 °C/min up to 380 °C, and held for 8 min. The total analysis time was 45 min.

The MS detector was operated in electron impact (EI) mode at 70 eV and 280 °C. The mass scan range was set from 29 to 1050 amu. The FID detector was set at a temperature of 400 °C with a hydrogen flow of 30 mL/min, an air flow of 400 mL/min, and a makeup flow (nitrogen) of 25 mL/min. The eluent from the column was split (1:2, splitter at a 25 kPa constant pressure) and simultaneously analyzed by MS for identification and FID for quantification.

Data processing was done with MassHunter Unknown Analysis (MSD Chemstation F.01.01.2317). Tentative identification of compounds was achieved by comparing the mass spectra with mass-spectral databases (NIST 17, Wiley10, and an in-house mass spectral library) after deconvolution of the chromatograms. Major compounds were confirmed by comparison to authentic standards or by NMR after isolation (see below).

Quantification was conducted using resveratrol as an internal standard with FID response factors that were calculated based on the “molar response factor” (MRF) equation described by de Saint Laumer et al.^21^

### 2.5 Isolation of the Compounds

The acetone seed extract was separated using a normal-phase silica gel column (Merck silica gel 60, 35–70 mesh) by successive elution with chloroform:methanol:acetone:formic acid (150:40:20:9, *v/v/v/v*). The acetone extract (250 mg) was dissolved in acetone (10 mL). Silica (50 mg) was added, and the solution was mixed gently with the solution. Acetone was evaporated, and silica with the extract was dry-loaded into the column (20 g of silica, diameter 2 cm). The ratio of extract/silica was 1:80 w/w. A fraction was collected every 2 mL. The purified fractions were monitored by high-performance thin-layer chromatography (CAMAG, Muttenz, Switzerland) using the same solvent. Three main fractions were obtained, including fraction 1 (piceatannol, *R*_f_ = 0.79), fraction 2 (epiafzelechin, *R*_f_ = 0.76), and fraction 3 (two epiafzelechin dimers, *R*_f_ = 0.37–0.40). Fractions with the same R_f_ were pooled, evaporated to dryness under a nitrogen stream, and stored at −20 °C before NMR analysis.

### 2.6 Structure Elucidation

All NMR spectra were recorded on a Bruker Avance II 400 (resonance frequencies: 400.13 MHz for ^1^H and 100.63 MHz for ^13^C) equipped with a 5 mm N_2_-cooled cryo probe head (Prodigy) with z-gradients with standard Bruker pulse programs. The samples were dissolved in 0.6 mL of either methanol-*d*_4_ (99.8% D), acetone-*d_6_* (99.8% D) or DMSO-*d*_6_ (99.8% D). Chemical shifts are given in parts per million, referenced to residual solvent signals (MeOD: δ_H_ 3.31 ppm, δ_C_ 49.0 ppm; acetone: δ_H_ 2.05 ppm, δ_C_ 29.8 ppm, DMSO: δ_H_ 2.49 ppm, δ_C_ 39.6 ppm). ^1^H NMR data were collected with 32k complex data points and apodized with a Gaussian window function (lb = −0.3 Hz and gb = 0.3 Hz) prior to Fourier transformation. The ^13^C spectrum with WALTZ16 ^1^H decoupling was acquired using 64k data points. Signal-to-noise enhancement was achieved by multiplication of the FID with an exponential window function (lb = 1 Hz). All two-dimensional experiments were performed with 1k × 256 data points, while the number of transients and the sweep widths were optimized individually. The HSQC experiment was conducted using an adiabatic pulse for inversion of ^13^C and GARP-sequence for broadband ^13^C-decoupling, optimized for ^1^ *J*_(CH)_ = 145 Hz. The long-range C, H coupling constant was set to 8 Hz in the HMBC spectra.

The optical rotation of the compound was determined based on its rotation in polarized light. 2 mg of purified epiafzelechin (90% purity according to ^1^H NMR) was dissolved in methanol (Merck, Germany), and then, the optical rotation was measured on an MCP 100 polarimeter (Anton Paar, Austria).

### 2.7 Compound Identification

#### 2.7.1 Piceatannol (**10**)

^1^H NMR (400 MHz, acetone-*d*_6_): δ 7.07 (d, 1H, *J* = 2.0, H-2), 6.95 (d, 1H, *J* = 16.4, H-7), 6.90 (dd, 1H, *J* = 8.1, 2.0, H-6), 6.82 (d, 1H, *J* = 16.4, H-8), 6.80 (d, 1H, *J* = 8.1, H-5), 6.52 (d, 2H, *J* = 2.2, H-10, H-14), 6.26 (t, 1H, *J* = 2.2, H-12); ^13^C NMR (100 MHz, acetone-*d*_6_): δ 159.6 (C-11, C-13), 146.15 (C-3/C-4), 146.12 (C-3/C-4), 140.8 (C-9), 130.7 (C-1), 129.4 (C-7), 126.9 (C-8), 119.9 (C-6), 116.2 (C-5), 113.8 (C-2), 105.6 (C-10, C-14), 102.6 (C-12). The NMR assignments were consistent with ^1^H, ^13^C, HSQC, and HMBC spectra. See Figures S1–S5 for the spectra. The recorded trimethylsilylated ion at *m*/z 532.2 agreed with that of the derivatized piceatannol.

#### 2.7.2 Epiafzelechin (**9**)

^1^H NMR (400 MHz, methanol-*d*_4_): δ 7.32 (d, 2H, *J* = 8.6, H-2′, H-6′), 6.77 (d, 2H, *J* = 8.6, H-3′, H-5′), 5.94 (d, 1H, *J* = 2.4, H-6), 5.91 (d, 1H, *J* = 2.4, H-8), 4.87 (br.s, 1H, H-2), 4.18 (m, 1H, H-3), 2.88 (dd, 1H, *J* = 16.9, 4.9, H-4a), 2.74 (dd, 1H, *J* = 16.9, 3.0, H-4b); ^13^C NMR (100 MHz, methanol-*d*_4_): δ 158.05–157.4 (C-4′, C-8a, C-7, C-5, interchangeable assignments), 131.6 (C-1′), 129.3 (C-2′,C-6′), 115.8 (C-3′, C-5′), 100.0 (C-4a), 96.4 (C-6), 95.9 (C-8), 79.9 (C-2), 67.5 (C-3), 29.4 (C-4). The NMR assignments were consistent with ^1^H, ^13^C, HSQC, and HMBC spectra. See Figures S6–S10 for the spectra. The recorded trimethylsilylated ion at *m*/*z* 562.3 agreed with an ion of the derivatized epiafzelechin.

#### 2.7.3 Epiafzelechin Dimer (**11**)

^1^H NMR (400 MHz, methanol-*d*_4_, 328 K): δ 7.35–7.2 (m, 4H, H-2′, H-6′ ring B, ring E), 6.80–6.70 (m, 4H, H-3′, H-5′ ring B, ring E), 6.10–5.85 (br.s, 1H, H-6 ring A), 6.10–5.85 (br.s, 1H, H-8 ring A), 6.10–5.85 (br.s, 1H, H-6 ring D), 5.10 (br.s, 1H, H-2 ring C), 4.85 (br.s, H-2 ring F), 4.60 (br.s, H-4 ring C), 4.20 (br.s, 1H, H-3 ring F), 3.90 (br.s, 1H, H-3 ring C), 2.92 (dd, 1H, H-4a ring F), 2.74 (dd, 1H, H-4b ring F); ^1^H NMR (400 MHz, DMSO-*d*_6_, 343 K): δ 7.4–7.0 (m, 4H, H-2′, H-6′ ring B, ring E), 6.8–6.6 (m, 4H, H-3′, H5′ ring B, ring E), 5.95–6.60 (br.s, 1H, H-6 ring A), 5.95–6.60 (br.s, 1H, H-8 ring A), 5.74 (br.s, H-6 ring D), 5.10 (br.s, H-2 ring C), 4.85–4.70 (br.s, H-2 ring F), 4.44 (br.s, H-4 ring C), 4.25–4.0 (br.s, 1H, H-3 ring F), 3.71 (br.s, 1H, H-3 ring C), 2.75–2.65 (m, 1H, H-4 ring F), 2.45–2.3 (m, 1H, H-4 ring F); the NMR assignments were consistent with ^1^H, ^13^C, HSQC, and HMBC spectra. See Figures S11–S16 for the spectra. The molecular weight of the trimethylsilylated dimer exceeded the recorded range of the MS detector. The compound detected by LC-MS (Agilent 1100) had a molecular ion at *m*/*z* 545.1, which agreed with that of an anion with a single charge.

### 2.8 Statistical Analysis

Data were analyzed with OriginPro 2023b software (OriginLab Corporation, Northampton, MA, USA). One-way analysis of variance (ANOVA) and Tukey test were used to determine the difference of means (*n* = 3). Differences were considered statistically significant at *p* < 0.05.

## 3 Results and Discussion

### 3.1 Characteristic of Seedy Banana Fruit

The fruits of 3 varieties of seedy banana (Musa itinerans, Musa acuminata, and Ensete glaucum) at the green and ripening stages were investigated (Figure 1). First, the weight proportions of seeds, peel, and pulp after freeze-drying were determined (Figure 2). Between varieties, the weight portions of peel and pulp were significantly different (*p* < 0.001 and *p* = 0.004, respectively), whereas the weight proportions for seed did not show a significant difference (*p* = 0.073). The fraction of peel in the two Musa species ranged from 15.6% to 17.3% of the total dried fruit weight, approximately three times that in Ensete (6.05%). Correspondingly, Ensete showed the largest proportion of pulp (46.4%), compared to Musa itinerans with a pulp portion of 30.8–31.7%.

**Figure 2:**
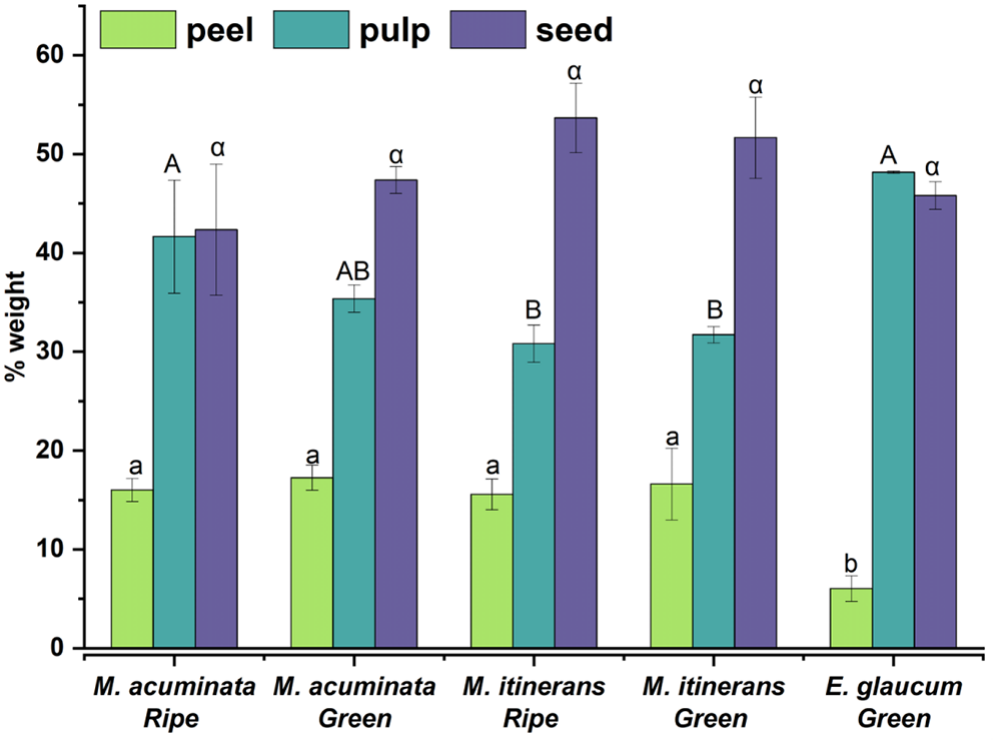
Weight fraction of fruit parts of the investigated seedy banana varieties. Mean ± SD (*n* = 3). Bars carrying different letters for a given part (peel, pulp or seed) are significantly different (*p* < 0.05).

The seed fraction of both green and ripe Musa itinerans was 51.7% and 53.7% of the dry weight, respectively, higher than that of Musa acuminata and Ensete glaucum at 42.3–47.4%. However, these differences were not statistically significant (*p* = 0.073). The seed fraction of all seedy bananas was higher than that of other valuable seed fruits, such as pomegranate, of which 4.0–10.0% of the fruit is seeds,^22^ or passion fruit (about 12% seeds).^23^ The high fraction of seeds makes seedy bananas less attractive to consumers as food compared with commercial dessert bananas, but seedy bananas might be a good source for potent bioactive compounds that are concentrated in the seeds.

### 3.2 Characterization of Main Compounds by GC-MS

Seeds were extracted, and the combined extracts were subjected to GC analysis after derivatization (Figure 3 and Table 1), according to a literature protocol developed in our lab. In general,MusaandEnsete varieties showed similar extractive profiles, with 11 major peaks found in all three varieties. One component, gallocatechin, was found in Ensete only and was not detected inMusa. These peaks represent nine different compounds from the classes of carbohydrates, fatty acids, and phenolics (Figure 4, Table 1).

**Figure 3:**
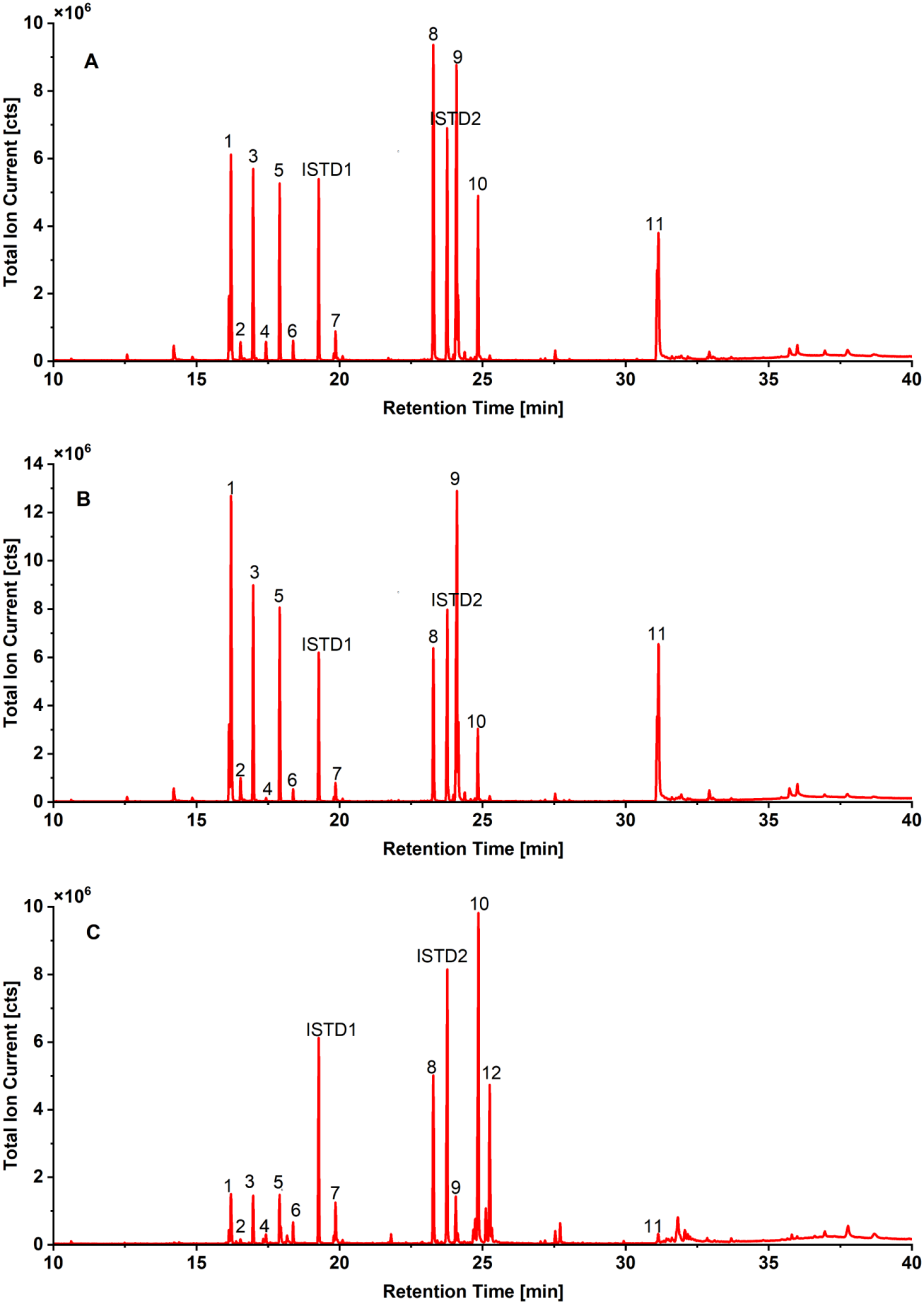
Gas chromatograms of acetone extracts of seeds of ripe fruits (A: Musa acuminata, B: Musa itinerans), and green fruits (C: Ensete glaucum). 1, 2: fructose; 3, 4: glucose; 5: sorbitol/mannitol; 6: palmitic acid; 7: oleic acid; 8: sucrose; 9: (+)-epiafzelechin; 10: piceatannol; 11: epiafzelechin dimers; 12: gallocatechin; ISTD1: margaric acid; ISTD2: resveratrol.

**Table 1:** Compounds detected by GC-MS in the Seeds of Musa acuminata, Musa itinerans (*Musas*), and Ensete glaucum (Ensete)­^a^

Peaks	Retention time (min)	Compound name	Derivatization Formular	Found in
*Sugar and organic acid*				
1	16.20	Fructopyranose^2^	C_21_H_52_O_6_Si_5_	*Musas*, Ensete
2	16.54	Fructopyranose^2^	C_21_H_52_O_6_Si_5_	*Musas*, Ensete
3	16.98	Glucopyranose^2^	C_21_H_52_O_6_Si_5_	*Musas*, Ensete
4	17.91	Glucopyranose^2^	C_21_H_52_O_6_Si_5_	*Musas*, Ensete
5	17.43	Alcohol sugar (sorbitol or mannitol)^3^	C_13_H_31_O_3_Si_3_	*Musas*, Ensete
8	23.28	Sucrose^2^	C_36_H_86_O_11_Si_8_	*Musas*, Ensete
*Fatty acid*				
6	18.38	Palmitic acid (16:0)^3^	C_19_H_40_O_2_Si	*Musas*, Ensete
7	19.86	Oleic acid (18:1)^3^	C_21_H_42_O_2_Si	*Musas*, Ensete
*Phenolic compounds*				
9	24.01	Epiafzelechin^1^	C_27_H_46_O_5_Si_4_	*Musas, Ensete*
10	24.84	Piceatannol^1^	C_26_H_44_O_4_Si_4_	*Musas*, Ensete
11	31.12	Epiafzelechin dimer^1^	C_54_H_90_O_10_Si_8_	*Musas*, Ensete
12	25.24	Gallocatechin^3^	C_33_H_62_O_7_Si_6_	Ensete

a 1) Identity was confirmed with NMR. 2) Identity was confirmed with an authentic standard. 3) Identity was assigned by the mass spectral library.

**Figure 4:**
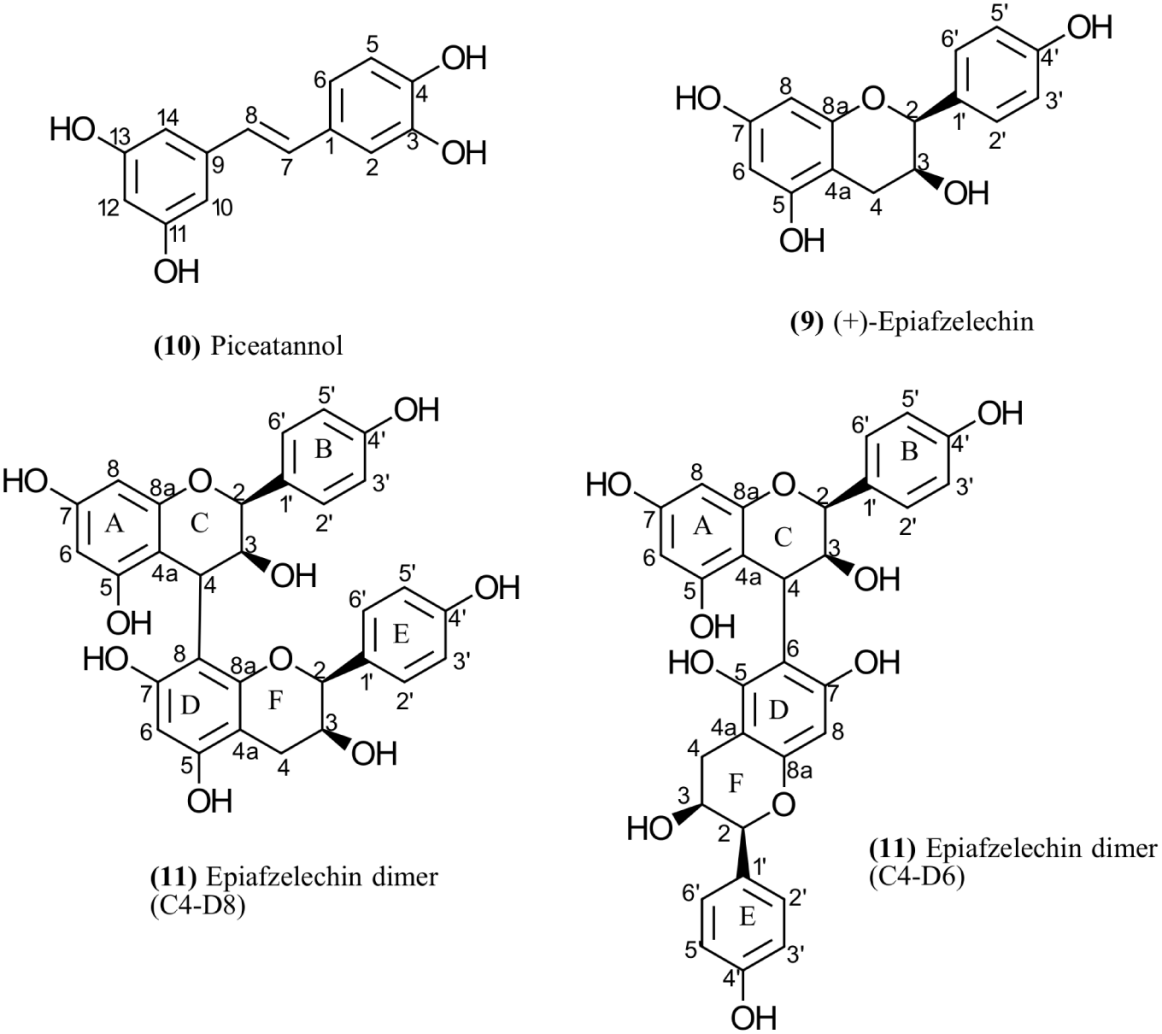
Structure of phenolic compounds in seedy bananas confirmed by NMR spectroscopy.

#### 3.2.1 Primary Metabolites

Five carbohydrates, including fructose, glucose, sugar alcohols (sorbitol or mannitol), and sucrose, were identified (peaks 1, 2, 3, 4, 5, and 8 in Figure 3). They were found in all three seedy banana varieties at both green and ripe maturity stages. These compounds are key primary metabolites and are, therefore, expected in all plants. Two fatty acids, palmitic acid (C16:0, peak 6 in Figure 3) and oleic acid (C18:1, peak 7 in Figure 3), were detected as well. For the investigated banana seeds, a low oil content of only 0.13% was determined by ASE extraction with hexane. Given the low oil content, the oil was not removed before extraction for the samples that were subjected to GC analysis, and the two major fatty acids (palmitic and oleic acids) were still present in this extract.

Some primary metabolites were identified in green seeds including amino acids (l-aspartic acid, lysine, serine, threonine, glutamic acid, and asparagine), organic acids (citric acid and malic acid), sugar alcohols (glycerol), and octadecenoic acid. These compounds were detected with match factors greater than 95% for the library search. They were found at lower concentrations in ripe seeds or were not detectable at all.

#### 3.2.2 Phenolic Compounds

Four major phenolic compounds were identified, with three of them present in all three varieties: piceatannol (peak 10, Figure 3), epiafzelechin (peak 9, Figure 3), and the dimer of epiafzelechin (peak 11, Figure 3). One phenolic, gallocatechin (peak 12, Figure 3), was found only inEnsete. Among the four phenolic compounds, piceatannol was detected as one of the most pronounced peaks across all varieties (peak 10, Figure 3). After a first assignment by a library search (match factor 93%), the identity of the compound was confirmed by NMR spectroscopy after purification by flash chromatography (Figures 4 and S1–S5). Piceatannol is a hydroxy derivative of resveratrol, which is a phenolic stilbene. It is a potent bioactive compound already found in grapes,^24^ passion fruit,^25^ Rhodomyrtus tomentosa seeds,^26^ and Gewang seeds.^27^ Piceatannol is also considered a powerful antioxidant with high activity at neutralizing reactive oxygen species,^28^ anti-inflammatory effects,^29^ and anticancer effects.^30^ Additionally, it exhibited various other biological activities, including protection of the skin against ultraviolet B irradiation,^31,32^ inhibition of melanogenesis,^33^ reducing the risk of cardiovascular diseases,^34,35^ and lowering of cholesterol levels.^36^

The second major phenolic compound in both Musa and Ensete was epiafzelechin (Figure 4). The compound was not found in the GC databases. After isolation, the purified fraction gave NMR spectra typical of epiafzelechin, a flavan-3-ol (Figures S6–S10). According to the positive optical rotation, its configuration was determined to be (+)-epiafzelechin. (+)-Epiafzelechin is a flavan-3-ol, which has been detected in several different foods, such as in green tea,^37^ in aerial parts of Celastrus orbiculatus,^38^ in the root bark ofCassia sieberiana,^39^ and in the seed extract of Musa balbisiana.^6^ Similar to other flavonoids, epiafzelechin possesses antioxidant and antiinflammatory activities,^38,39^ osteoprotective activity,^40^ and in vitro activity against cancer cells.^41^

Peak 11 (Figure 3) was found in the seeds of all investigated varieties. It consists of two nearly coeluting isomeric compounds, which exhibited the same fragment ions in their mass spectra. A database search was inconclusive. After isolation by flash chromatography, NMR spectra of the purified compound were recorded in methanol-*d*_4_ at 328 K and in DMSO-*d*_6_ at 343 K. The structures derived from the spectra showed dimers of epiafzelechin. Two broad peaks were observed in DMSO-*d*_6_ at chemical shifts of 5.88–5.94 ppm and 5.62–5.72 ppm, respectively, suggesting these coeluting compounds are a mixture of two isomers with carbon linkages C4-D8 and C4-D6, respectively (Figures 4 and S11–S16). The two epiafzelechin moieties in these dimers are linked by interflavonoid bonds and are hindered from free rotation so that atropisomers exist.^42^ This resulted in broad peaks in the ^1^H NMR spectra.^43^ In addition, the structure of these epiafzelechin dimers was confirmed by cleavage of the interflavonoid linkage in two different experimental approaches: hydrolysis with trifluoroacetic acid (TFA, 0.15% at 80 °C, 30 min) and thiolysis (benzyl mercaptan, 5%, and TFA, 0.4%, 40 °C, 120 min). GC-MS analysis of the degradation products showed that epiafzelechin was the only reaction product when incubated with TFA (Figure S17). The products of thiolytic cleavage were epiafzelechin and epiafzelechin benzyl thioether (Figure S18), which additionally confirmed the structure of the compounds as dimers of epiafzelechin. Epiafzelechin dimers belong to the proanthocyanidin group of phenolic compounds with various biological activities, such as antioxidant, cardioprotective, lipid-lowering, antiobesity,^44–46^ and neuroprotective activities.^47,48^ An anticancer effect is reported for proanthocyanidins both in vitro and in vivo.^49^

Peak 12 was identified as gallocatechin by a mass spectral search (98% match factor) and by comparison with fragmentation patterns reported in the literature.^50^ Potentially, this peak could also be epigallocatechin, since the fragmentation patterns are very similar and isolation by flash chromatography was not achieved. Gallocatechin was found to be a major component in Ensete but was not detected in *Musas*. Gallocatechin was also reported in the peel and pulp of the commercial banana Musa Cavendish at 158 mg/100 g DW and 29.6 mg/100 g DW, respectively.^51^ This compound is also detected in tea leaves.^52^

A comparison of the acetone extract and subsequent methanol extract showed that practically all of the epiafzelechin and its dimers were found in the acetone extracts. For piceatannol, however, the extraction efficiency was much higher with methanol, and the methanolic extract contained 70% of the total collected amount of piceatannol. The main phenolics might, therefore, be fractionated already during the extraction step by the choice of solvents.

### 3.3 Influence of Maturity and Variety on Main Bioactive Compounds in the Seed

The simultaneous detection by mass spectrometry and flame-ionization detection (FID) allowed us to determine the concentrations of phenolic compounds in the seeds. For this purpose, the response factor of the FID signal was estimated from the elemental composition using the combustion enthalpy. From this, the concentration of an identified compound can be determined in relation to an internal standard.^53^ The concentration of the major compounds was calculated using an equation described by de Saint Laumer et al.^21^ The obtained content of bioactive compounds in banana seeds is summarized in Figure 5.

**Figure 5:**
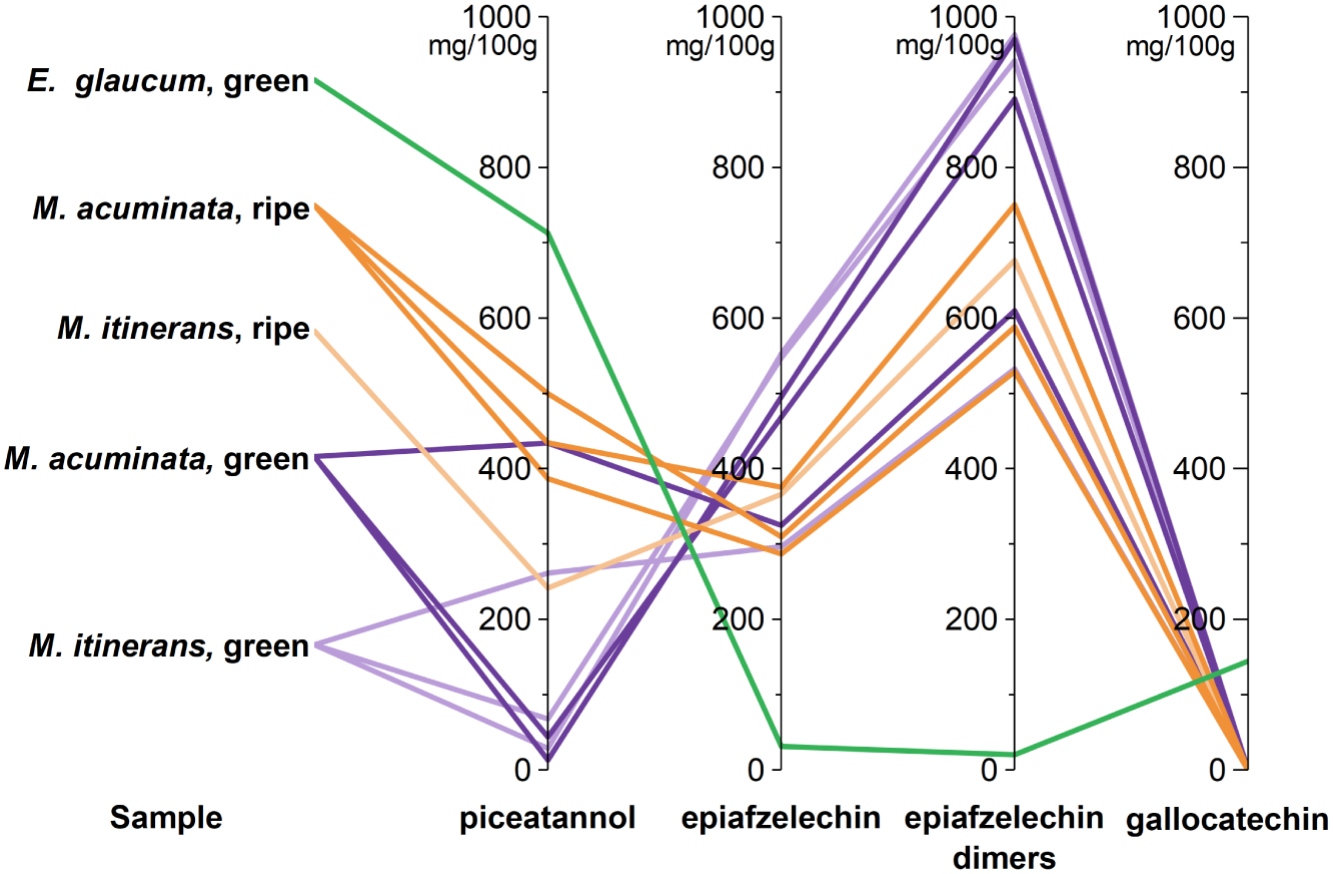
Overview of the contents of major bioactive compounds in banana seeds.

Ensete glaucum had the highest level of piceatannol, while the concentration of epiafzelechin was the lowest among all the samples. In particular, piceatannol was found to be high in Ensete at 713 ± 71.9 mg/100 g of DW, followed by ripe Musa acuminata at 440 ± 56.7 mg/100 g of DW, and Musa itinerans at 242 ± 17.4 mg/100 g of DW. These are remarkable contents. For comparison, piceatannol was reported in passion fruit seeds at 480 mg/100 g DW,^23^ in sugarcane at day 7 of incubation at 165.9 mg/100 g DW^54^ and in Rhodomyrtus tomentosa (seeds) at 230 mg/100 g DW.^26^ The piceatannol concentration in the seeds of ripe seedy bananas is among the highest reported levels. Its level in the seeds of Ensete was even 1.5 times higher than that in passion fruit seeds. In addition, the seed proportion of bananas was nearly 4 times that of passion fruit: 45.8% vs. 12%. The high portion of seeds in the fruit and the high content of piceatannol in the seeds make Ensete glaucum a promising candidate for the specific production of this bioactive phenolic compound. Likewise, the *Musas*are a source of epiafzelechin and its dimers.

The seeds of green *Musas* showed a larger variation in the concentrations of piceatannol than those of ripe seeds. In detail, the piceatannol content in the biological replication (three different bunches) of seeds of green Musa acuminata was 434, 44.2, and 13.7 mg/100 g of DW, respectively (RSD = 143%), and the values for seeds of green Musa itinerans were 262, 29.2, and 68.2 mg/100 g of DW (RSD = 104%). The biological replicates of ripe Musa accuminata were more consistent with piceatannol contents of 500, 387, and 435 mg/100 g of DW, respectively (RSD = 13%). A possible cause for the high variability observed for green bunches could be the difference in fruit maturity in addition to environmental factors. Piceatannol, a stilbene, is synthesized progressively during fruit development. Stilbenes are synthesized via the phenylpropanoid pathway, which is regulated by stilbene synthase genes, which are activated by specific developmental or environmental signals.^55^ In grapes, for example, stilbenes accumulate gradually during the ripening process.^56,57^ The seedy banana samples in this research were selected and harvested in nature based on the morphology of the fruit, which takes about 2–3 months for development and matures before reaching the ripening stage.^58^ Because the appearance of the green peel changes little until ripeness, it is hard to determine the exact developmental stage of the green fruit samples upon sampling. Our observations indicate an alteration of piceatannol during maturing, which would have to be confirmed in further studies.

Epiafzelechin had an inverse correlation with piceatannol concentration (Pearson correlation coefficient *r* = −0.91, *p* < 0.0001). Similarly, dimers of epiafzelechin also had a negative correlation with piceatannol (*r* = −0.89, *p* < 0.0001) and were strongly positively correlated with the monomer (*r* = 0.99, *p* < 0.0001). The samples with a high level of piceatannol thus exhibited low levels of epiafzelechin and its dimers, and vice versa. Most of the green Musa samples (purple lines in Figure 5) showed low piceatannol concentration at an average of 38.8 ± 23.2 mg/100 g of DW and gave the highest results for both epiafzelechin at 516 ± 40.7 mg/100 g of DW and epiafzelechin dimers at 945 ± 38.9 mg/100 g of DW, respectively. In contrast, ripe *Musas*(orange lines, Figure 5) and two of the green samples showed a low content of epiafzelechin and its dimers. This reduction of flavan-3-ol levels during ripening has been reported for grapes.^59^ This reduction is caused by the competition for substrates between stilbene synthase and chalcone synthase, which are the key enzymes of piceatannol and flavonoid synthesis, respectively.^57^ Another reason for the low flavan-3-ol levels could be an uncontrolled oxidation process.^59^ All in all, the seeds of both the green and ripe Musa varieties exhibited a high concentration of bioactive compounds.

Although Ensete and Musa are in the same Musaceae family, Ensete is considered different from Musa based on both morphological characteristics and molecular phylogenetics.^1^ Consequently, Ensete glaucum exhibited a different chemical profile (green line in Figure 5), with a high gallocatechin content of 145 ± 19.7 mg/100 g of DW, which was not detected in *Musas*. Ensete also contained the highest piceatannol level at 713 ± 71.9 mg/100 g of DW, and at the same time, the lowest levels of epiafzelechin and its dimers at 31.8 ± 4.1 and 20.8 ± 7.3 mg/100 g of DW, respectively. Because only one green bunch of Ensete glaucum could be harvested, the variation between the green seed and the ripe seed of Ensete glaucum has not been investigated. Nonetheless, the chemical composition in Ensete glaucum, as well as in the ten bunches of Musa in this research, can be seen as an initial justification for the use of seedy banana in folk medicine and a suggestion for further functional food applications. Ensete glaucum may be suitable for piceatannol exploitation, whereas the green seeds of Musa acuminata, and Musa itinerans may be more valuable as sources of flavan-3-ols. Since the phenolic composition of the *Musas* changes during maturation, the time of harvesting could potentially be used to obtain a feedstock rich in piceatannol or epiafzelechin, respectively.

### 3.4 Distribution of Phenolic Compounds in Ensete Seed

While Musa species had small seeds (diameter: 4–5 mm), Ensete glaucum had big seeds (diameter >1 cm) (Figure 6) with a high portion of seed coat. These seeds can be easily separated into two parts: seed coat and endosperm. The coat contributed 75% of the mass of the seed and the endosperm 25%, accordingly. These two parts were ground, and their phenolic content was determined separately (see Figure 6).

**Figure 6:**
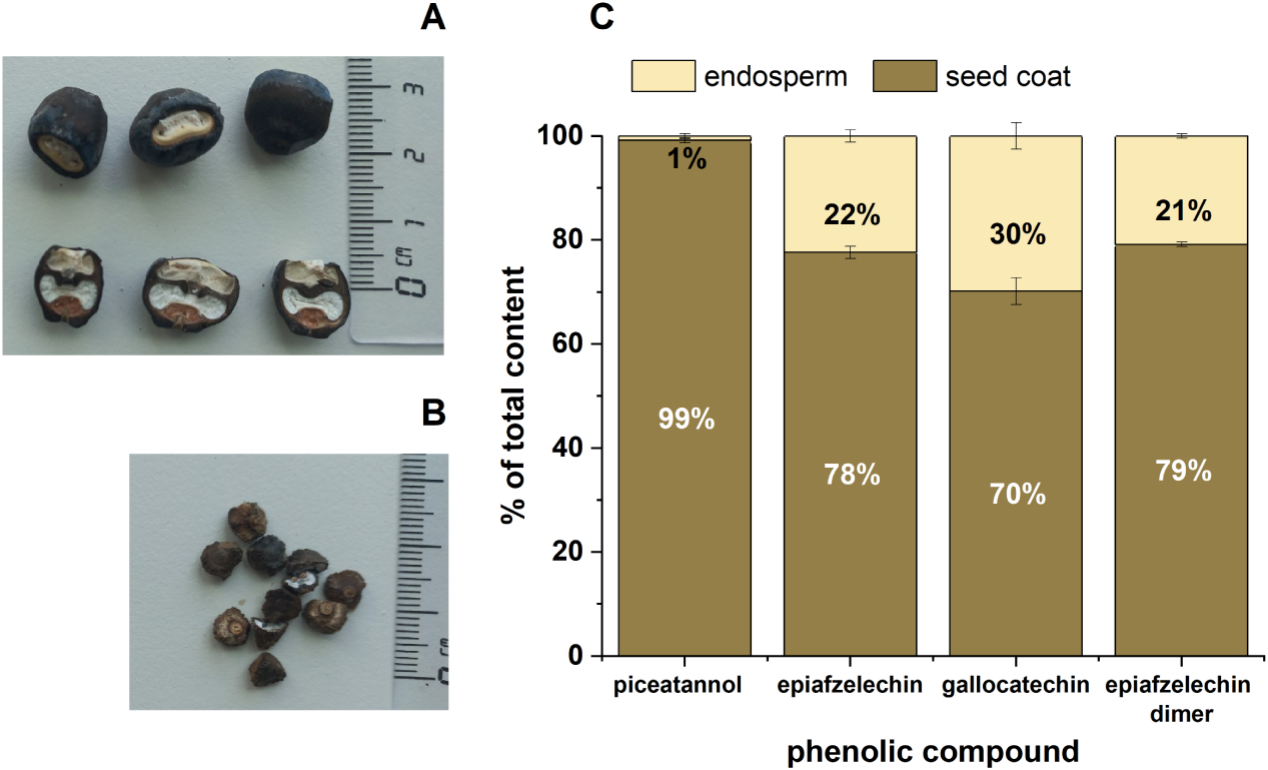
A) Seeds of Ensete. B) Seeds of Musa. C) Distribution of the phenolic compounds in the seed coats and endosperm of Ensete.

Among the three identified phenolic compounds, piceatannol showed the highest difference in content between the two parts, with 99% of piceatannol in the coat at a content equivalent of 942 ± 91.3 mg/100 g DW. Of the other determined flavonoids, 70–79% of the mass was found in the seed coat. A similar distribution was also reported in the seeds of red and black peanuts,^60^ lentils, and peas^61^ which had phenolic compounds mainly in the seed coat. That the phenols of interest are preconcentrated in the seed coat can facilitate the exploitation of bioactive compounds with higher yields.

The uneven distribution of the different phenolic compounds in the seeds of Ensete glaucum reflects a fundamental survival strategy of the plant.^62^ Flavonoids in seeds are crucial for increasing seed survival under severe conditions by defending against pathogens and environmental stress.^63,64^ Phenolic stilbenes act as antioxidants and have been described as a defense against fungal colonization in peanuts.^65^ In legume seeds, phenolics are involved in the defense mechanisms against pathogens and insect herbivores.^66^

Considering the notable content of phenolics in their seeds, seedy bananas could be cultivated as a source of food supplements and nutraceutical applications. In addition to biological applications, the banana plant can be a high-value feedstock for biorefinery scenarios.^67^ The yields of piceatannol and epiafzelechin depend on the choice of banana variety, extraction solvent, and fruit maturity. This could allow us to obtain feedstocks enriched with piceatannol or epiafzelechin, depending on the desired application. We hope that the results of this study will help to encourage farmers to cultivate seedy banana plants in the mountains and harvest fruits. This will contribute not only to preserving the biological heritage but also to increasing the application of natural bioactive compounds.

## Supplementary Material



## Notes

NMR spectra (1H, 13C, COSY, HSQC, HMBC) of piceatannol, epiafzelechin, and its dimer; gas chromatograms of the epiafzelechin dimer natively and after hydrolysis and thiolysis (PDF)
