# Supplementary material for: Seedy Banana – A Source of Stilbenes and Flavan-3-ols
Source: J Agric Food Chem. 2025 Jun 6;73(24):15103–12. doi: 10.1021/acs.jafc.5c01416 (PMC12186517; doi:10.1021/acs.jafc.5c01416)
Supplement: Supplementary file 1 [file jf5c01416_si_001.pdf]

Supporting Information

Seedy banana - a source of stilbenes and flavan-3-ols

Hoai Thi Tran[a,b], Markus Bacher[a], Stefano Barbini[c], Thomas Rosenau[a] & Stefan Böhmdorfer[a,*]

[a]*University of Natural Resources and Life Sciences, Vienna, Institute of Chemistry of Renewable Resources, Konrad-Lorenz-Straße 24, A-3430 Tulln, Austria*

[b]*Vietnam National University of Agriculture, Faculty of Food Science and Technology, 100 000 Hanoi, Vietnam*

[c]*NATEX Prozesstechnologie GesmbH, Werkstrasse 7, 2630 Ternitz,Austria*

*) stefan.boehmdorfer@boku.ac.at

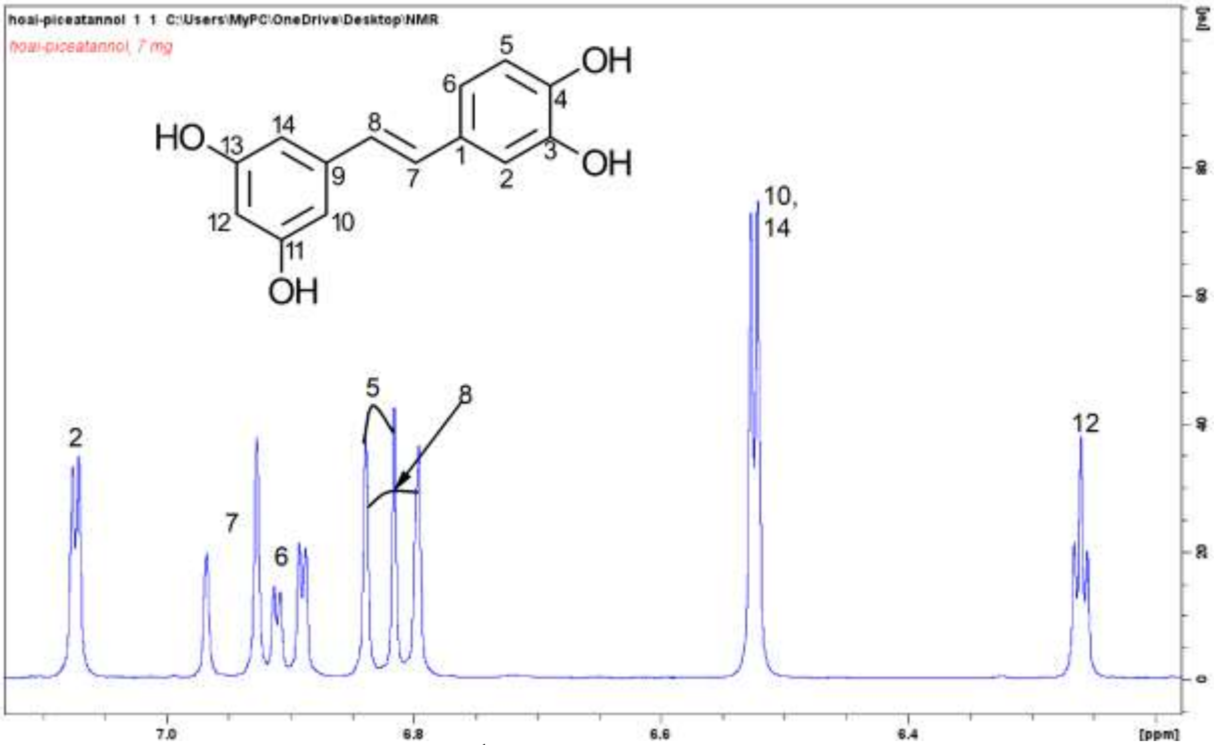

Figure S1: An expanded region of the ^{1}H NMR spectrum of piceatannol (400MHz).

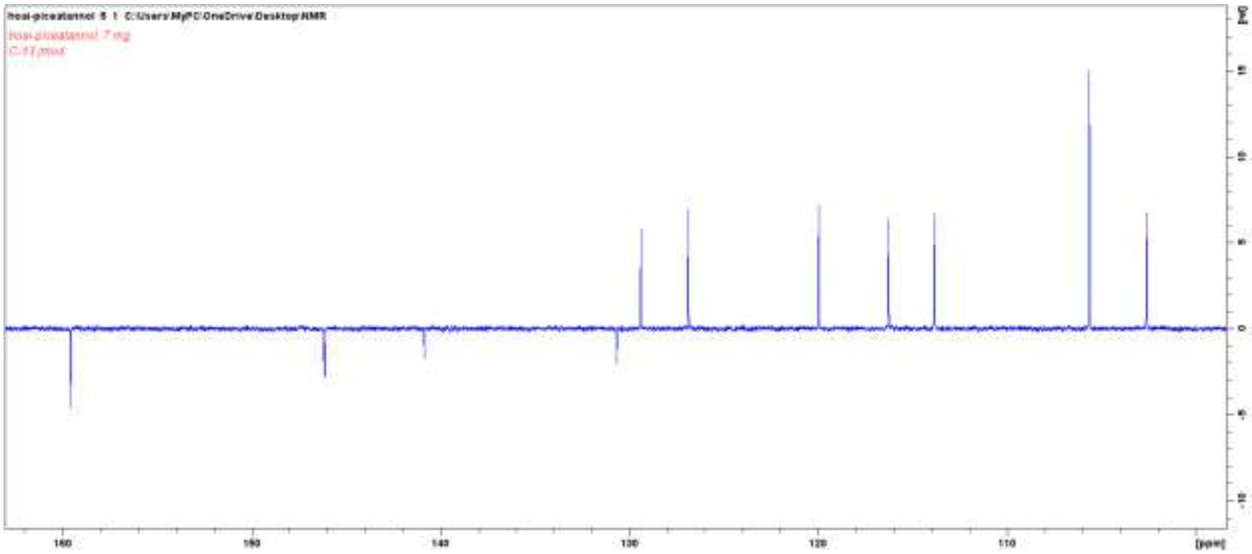

Figure S2. An expanded region of the ^{13}C NMR spectrum of piceatannol (100MHz).

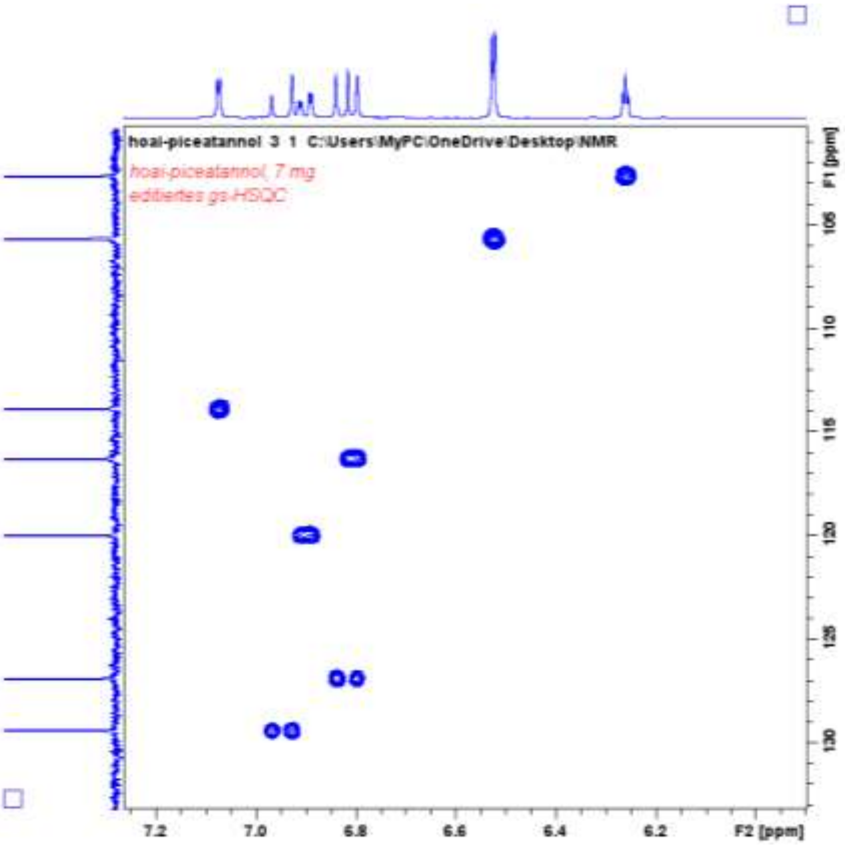

Figure S3. An expanded region of ^{1}H-^{13}C HSQC NMR spectrum of piceatannol

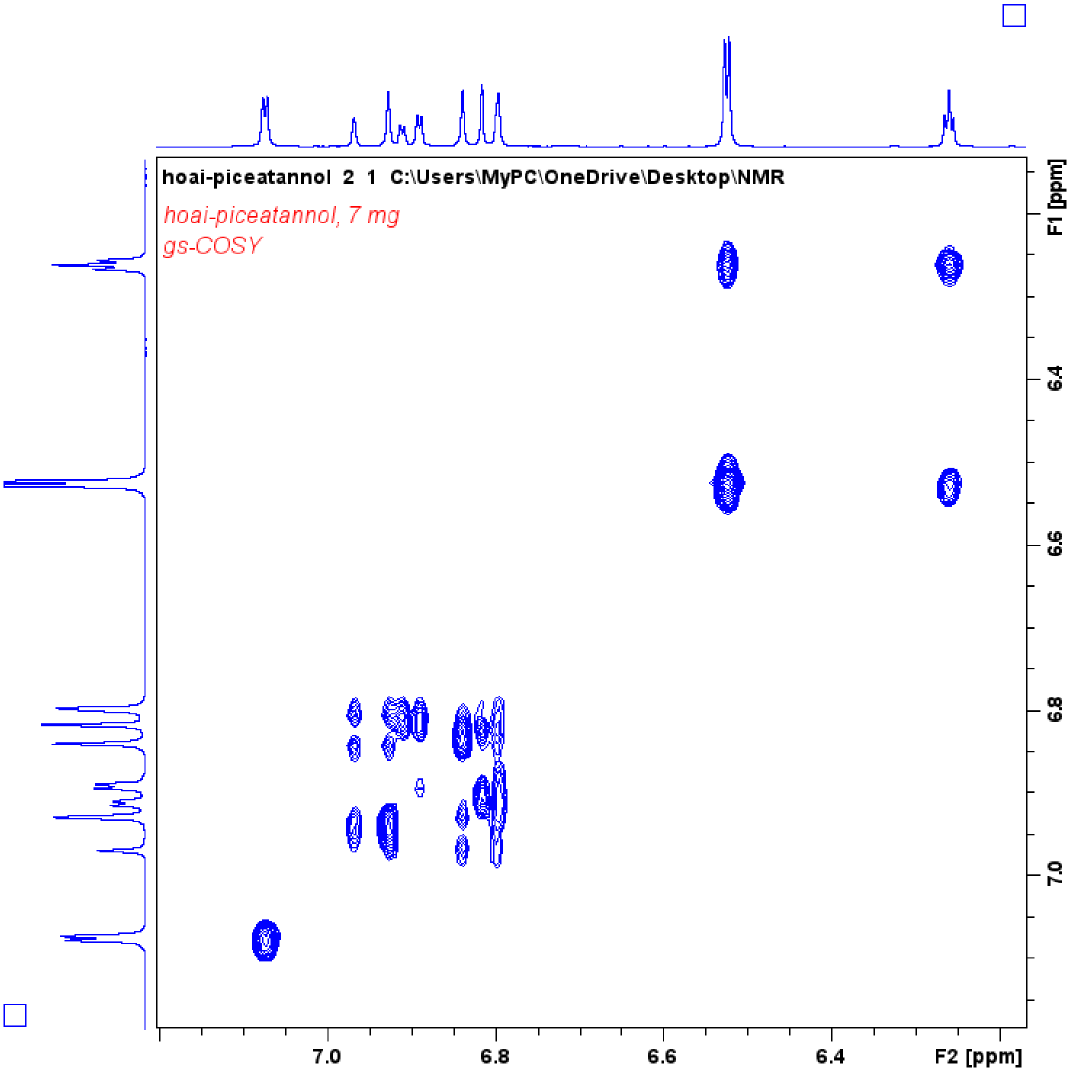

Figure S4. An expansion of the ^{1}H-^{1}H COSY NMR of piceatannol

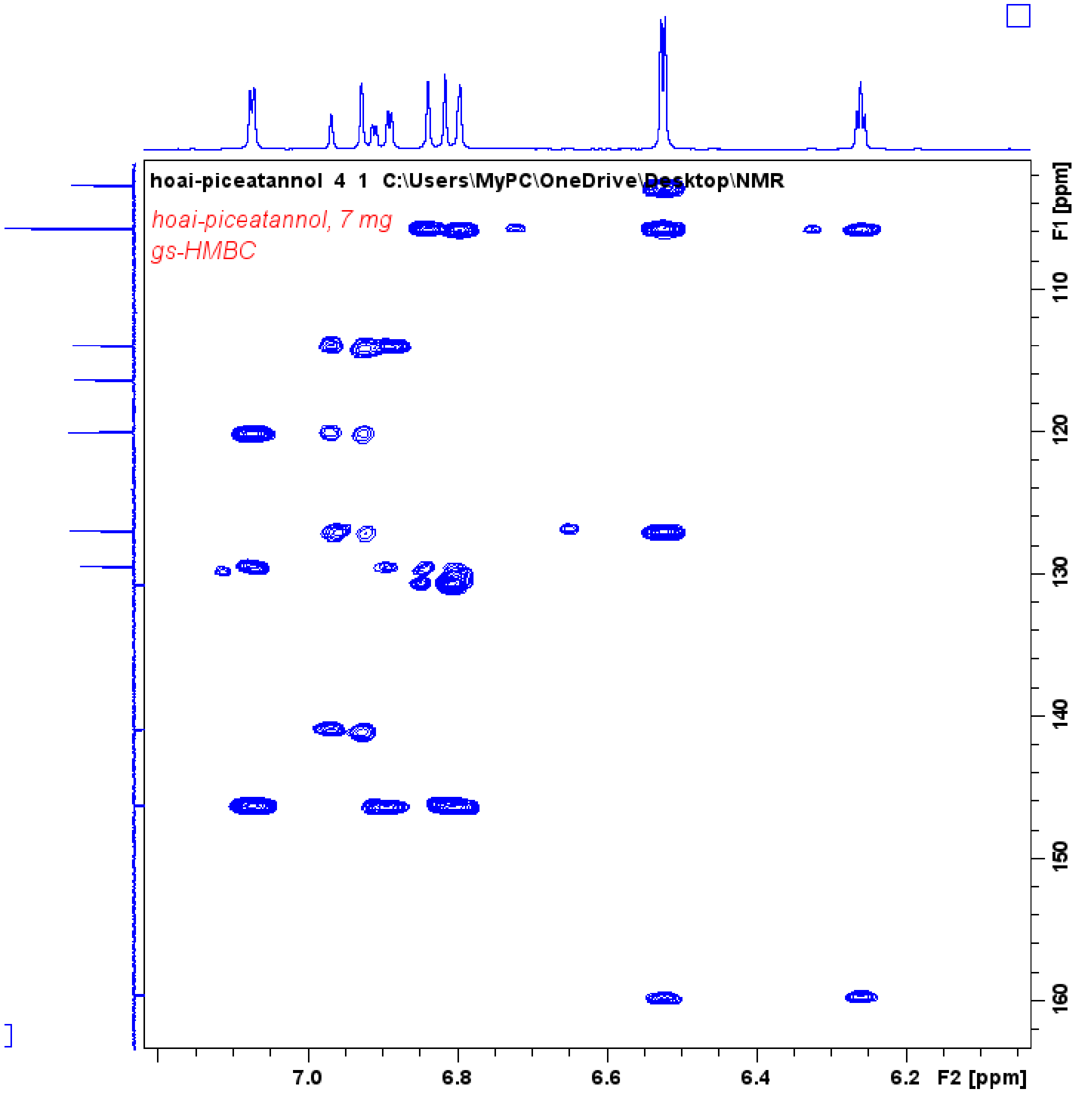

Figure S5. An expansion of the ^{1}H-^{13}C HMBC NMR of piceatannol

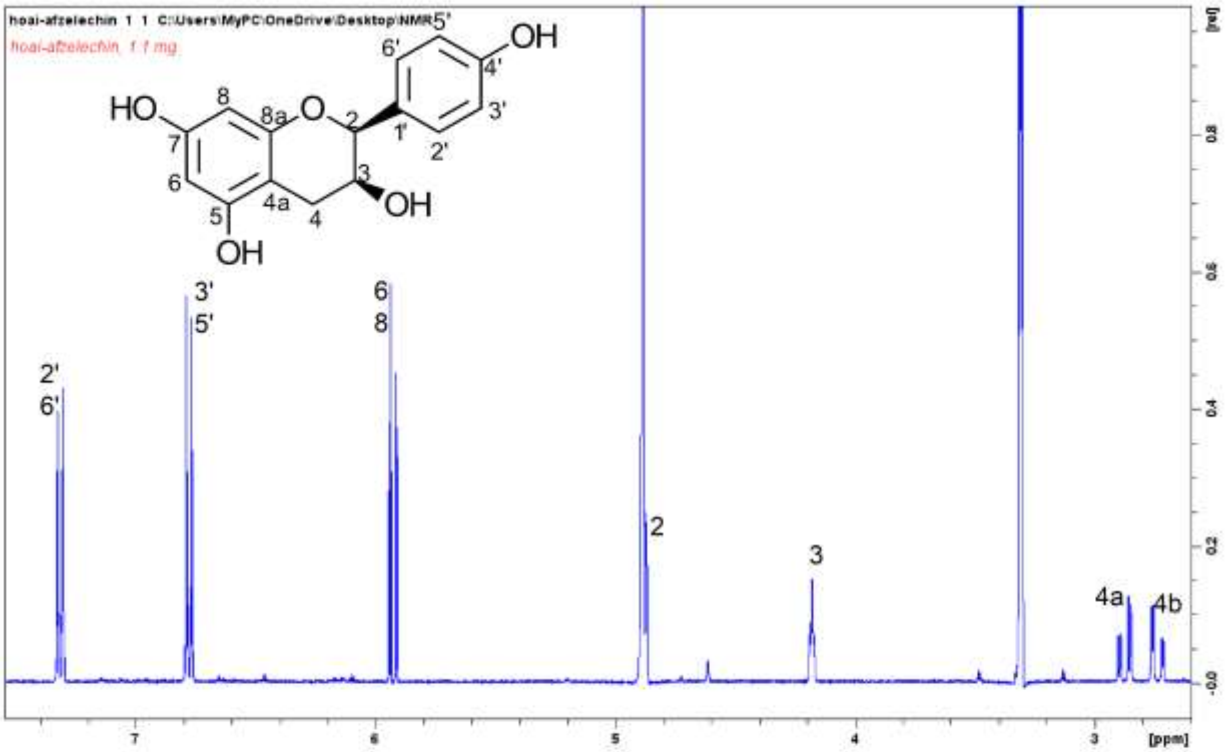

Figure S6. An expanded region of the ^{1}H NMR spectrum of epiafzelechin

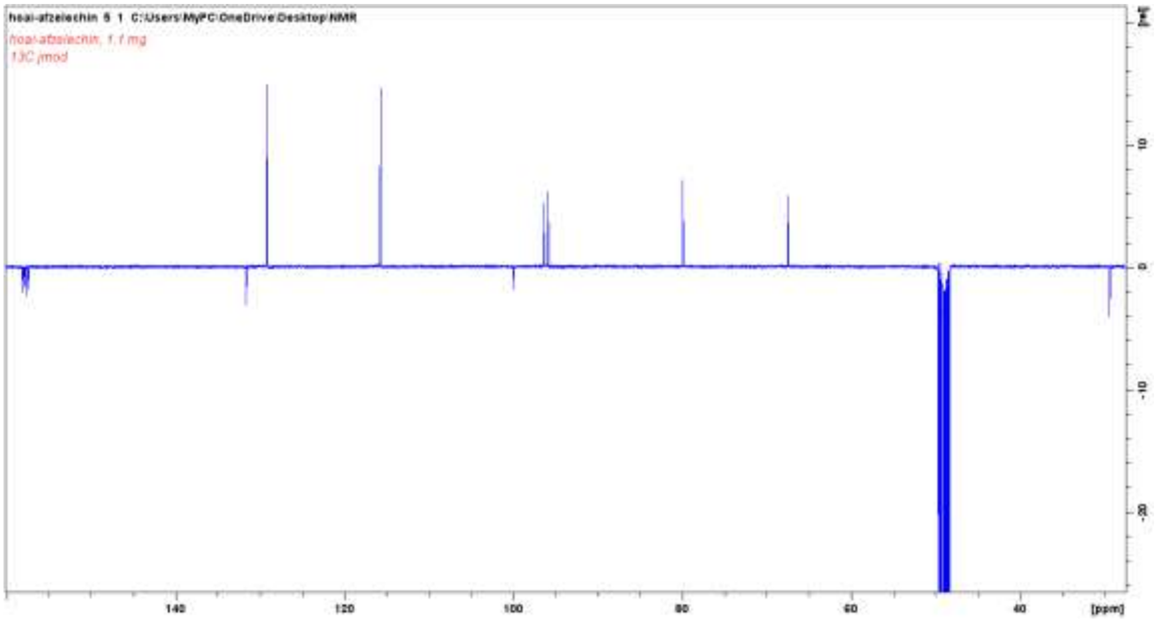

Figure S7: An expansion of the ^{13}C NMR spectrum of epiafzelechin

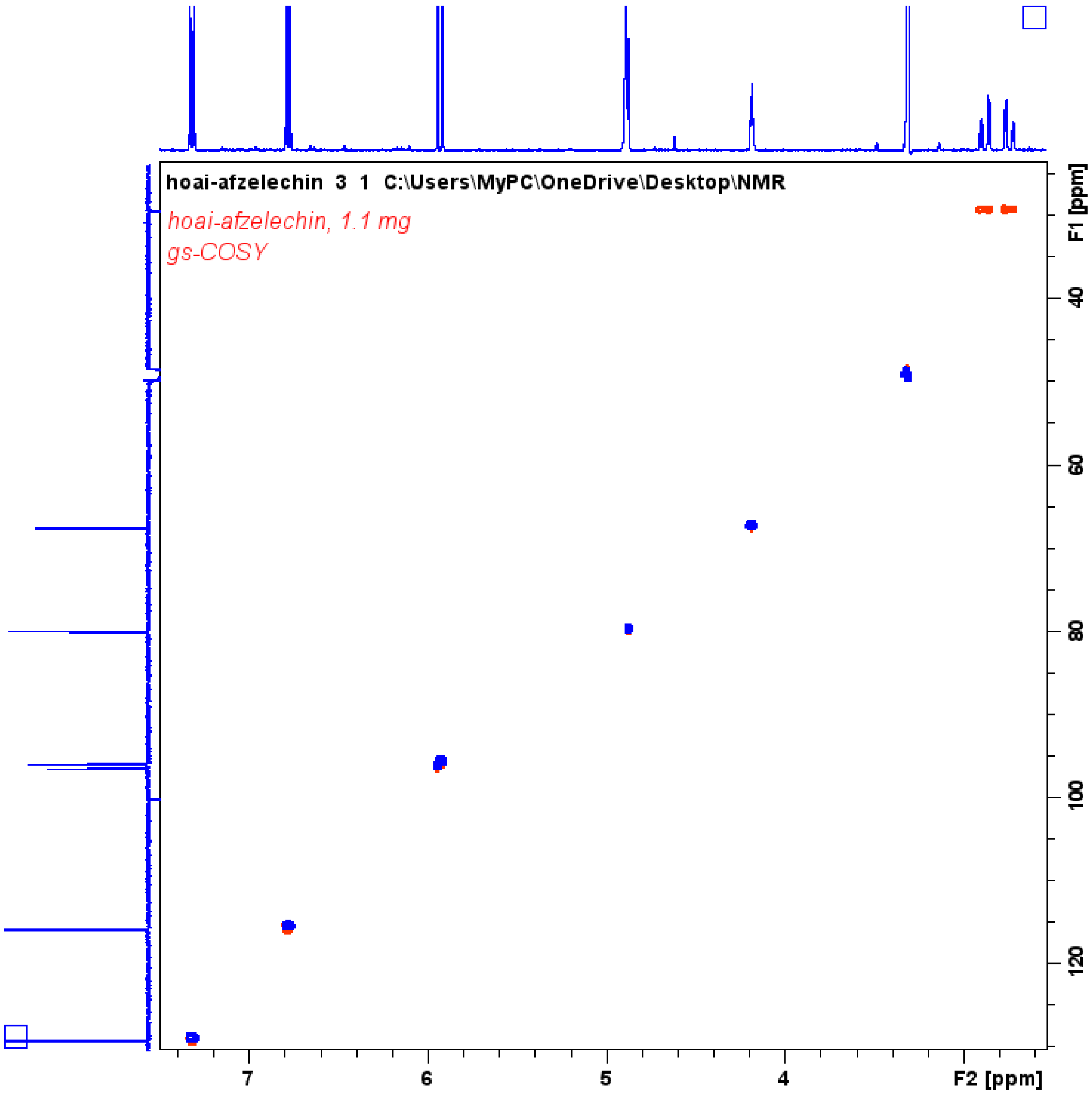

Figure S8. An expansion of the ^{1}H-^{13}C HSQC NMR spectrum of epiafzelechin

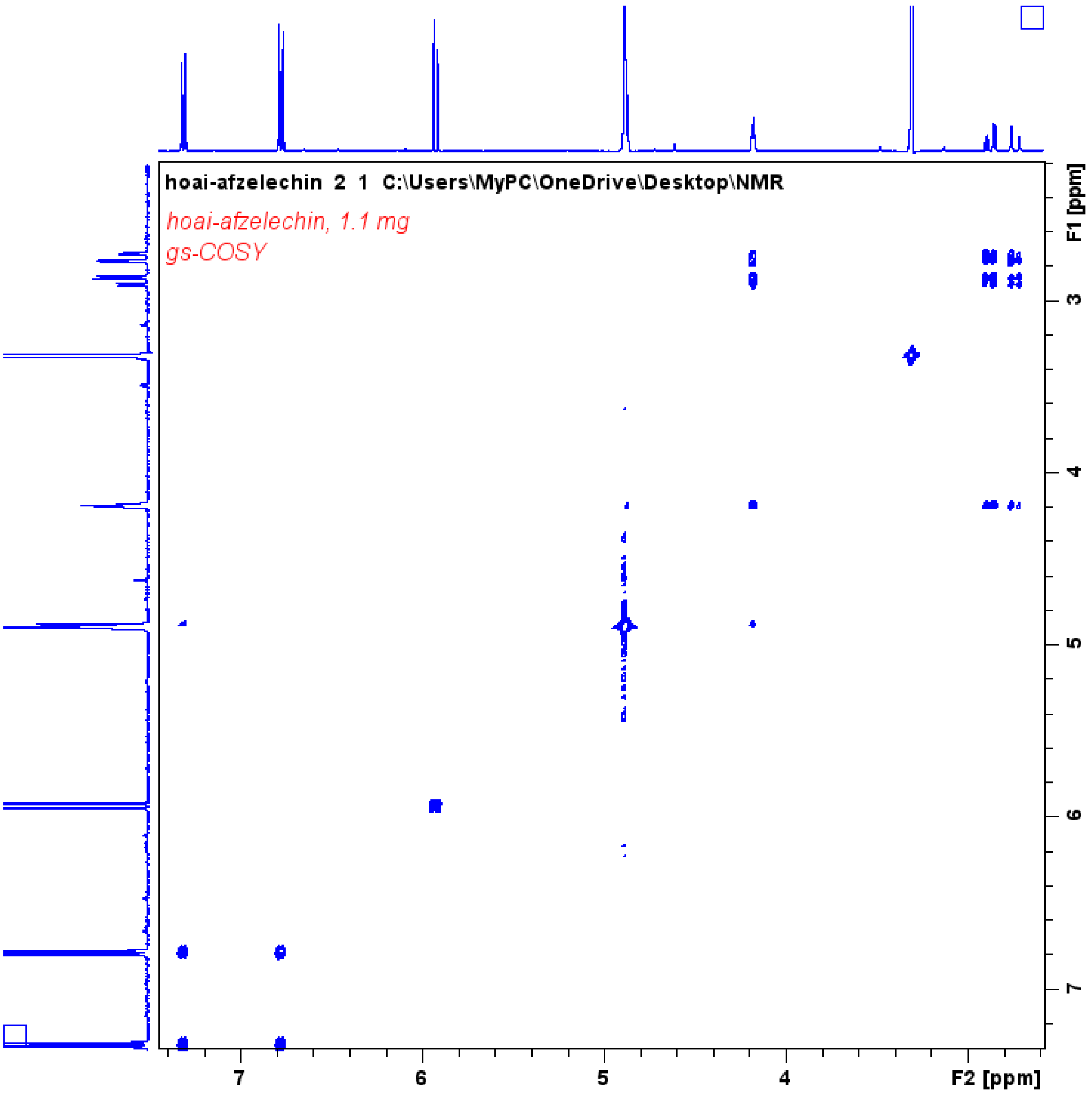

Figure S9. An expansion of the ^{1}H-^{1}H COSY NMR spectrum of epiafzelechin

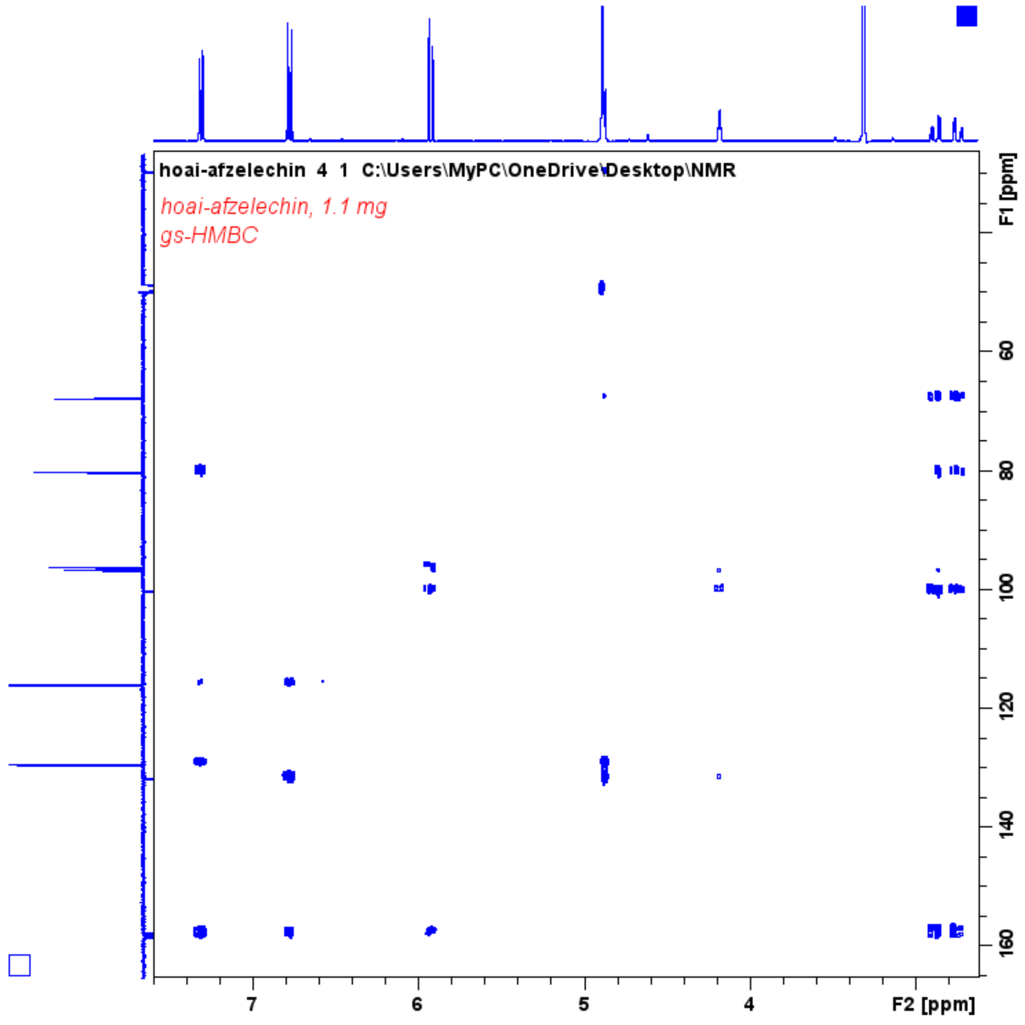

Figure S10. An expanded region of the ^{1}H-^{13}C HMBC NMR spectrum of epiafzelechin

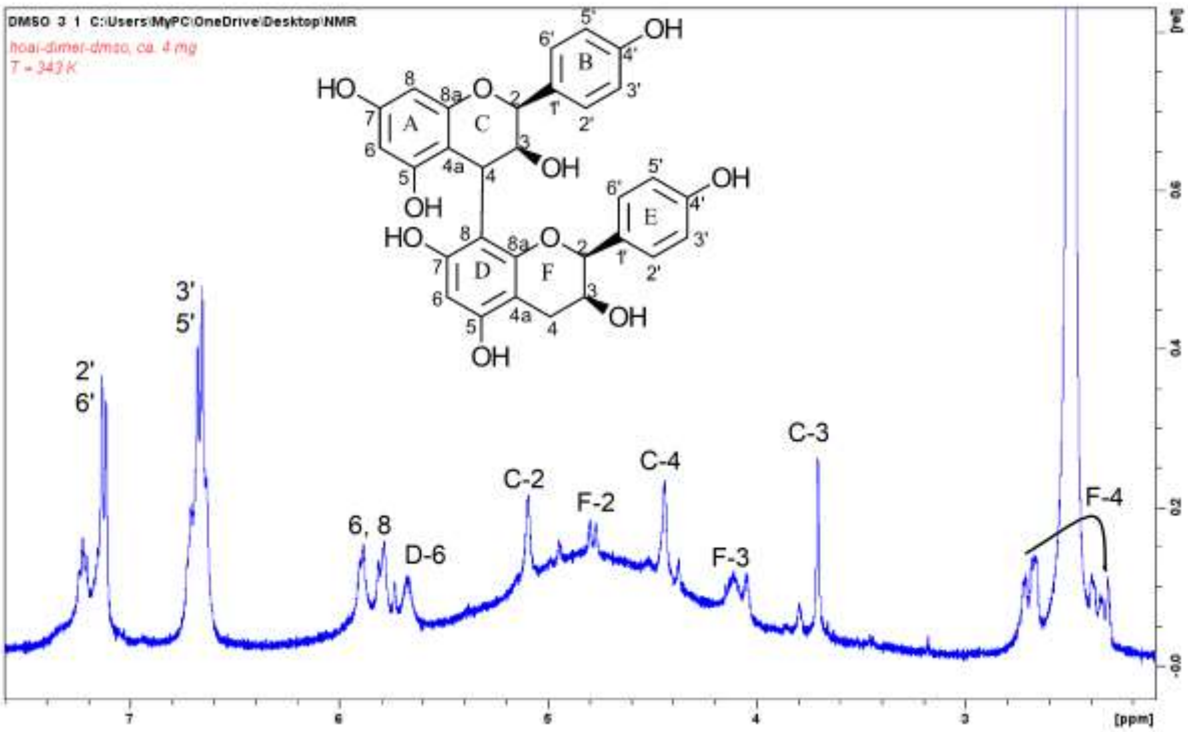

Figure S11. An expanded region of the ^{1}H NMR spectrum of epiafzelechin dimers (DMSO-*d6*)

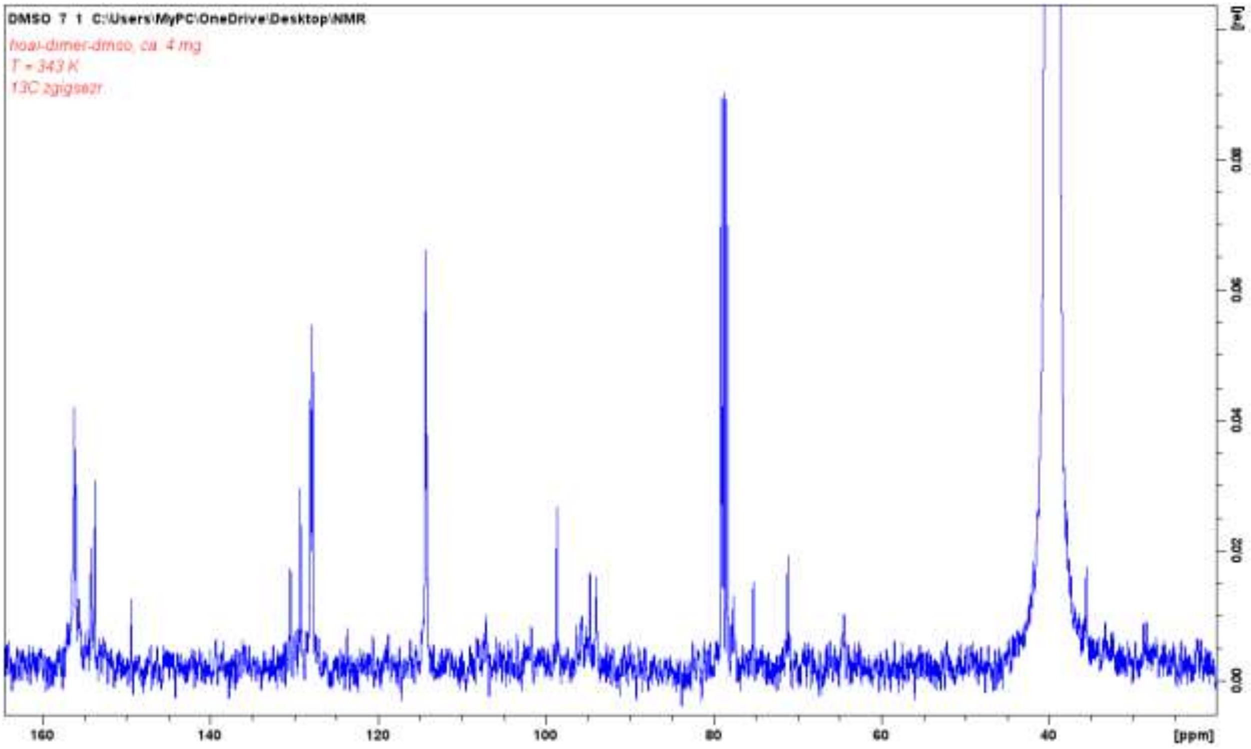

Figure S12: An expansion of the ^{13}C NMR spectrum of epiafzelechin

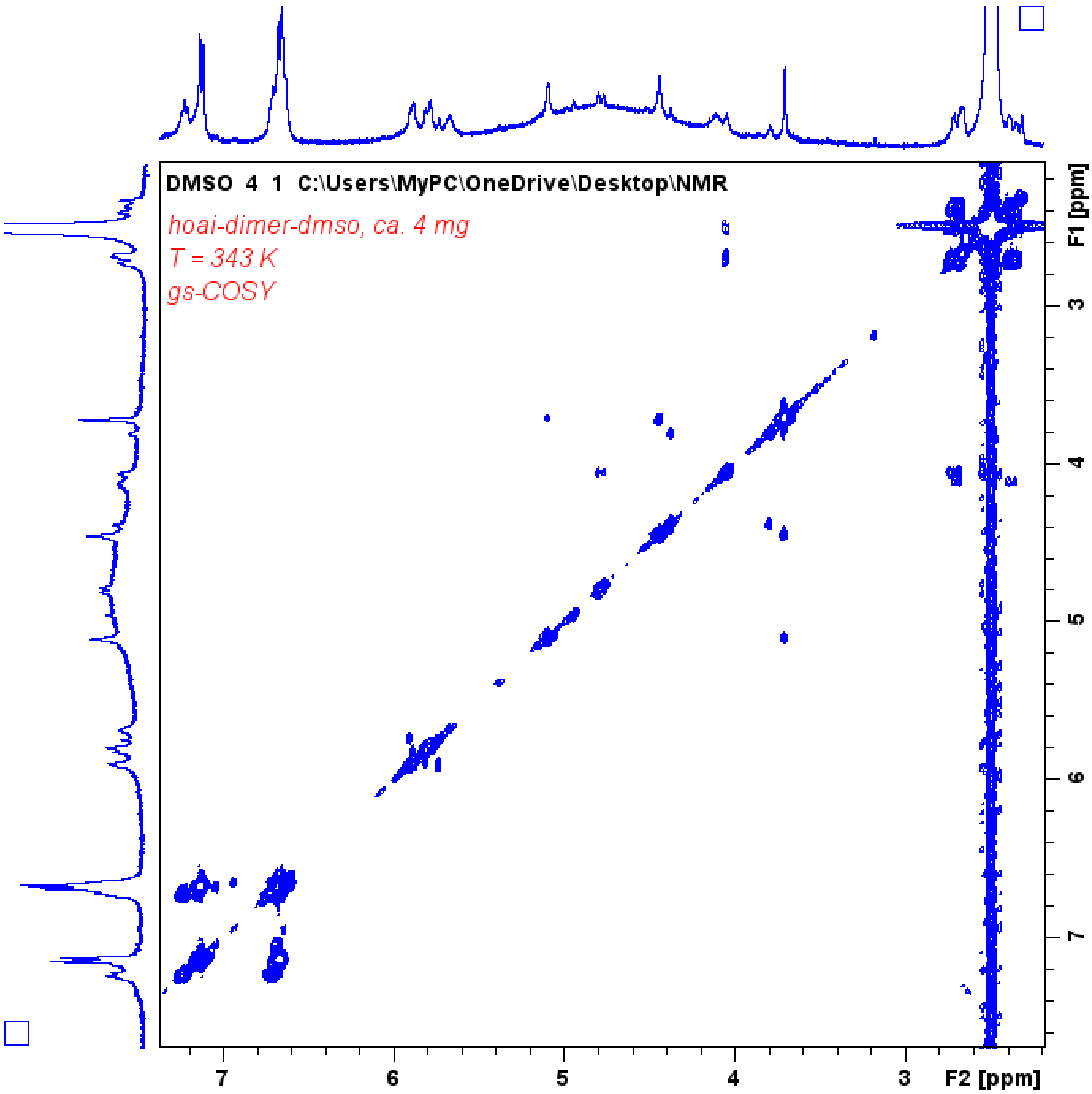

Figure S13. An expansion of the ^{1}H-^{1}H COSY NMR spectrum of epiafzelechin dimers

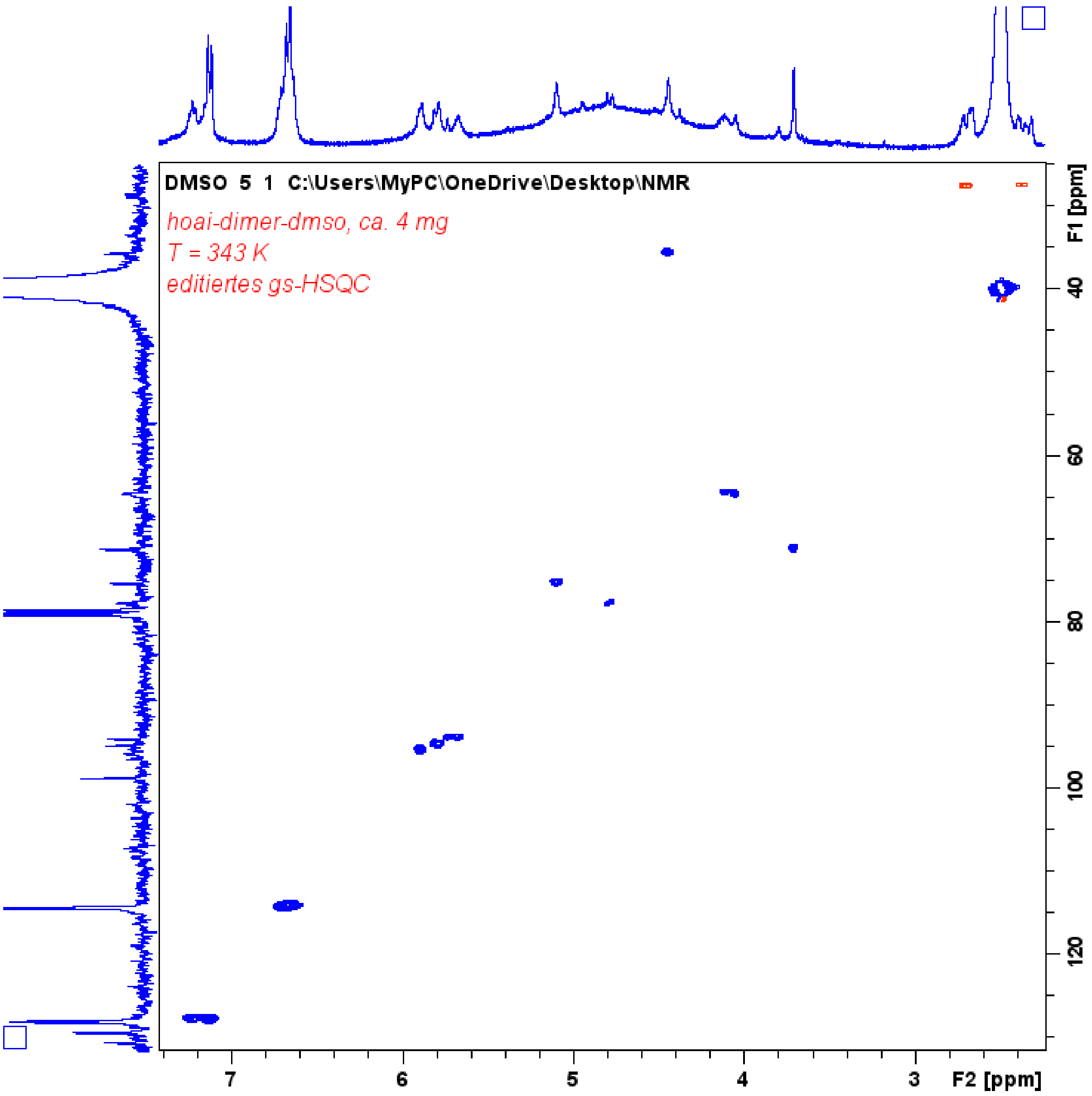

Figure S14. An expanded region of the ^{1}H-^{13}C HSQC NMR spectrum of epiafzelechin dimers

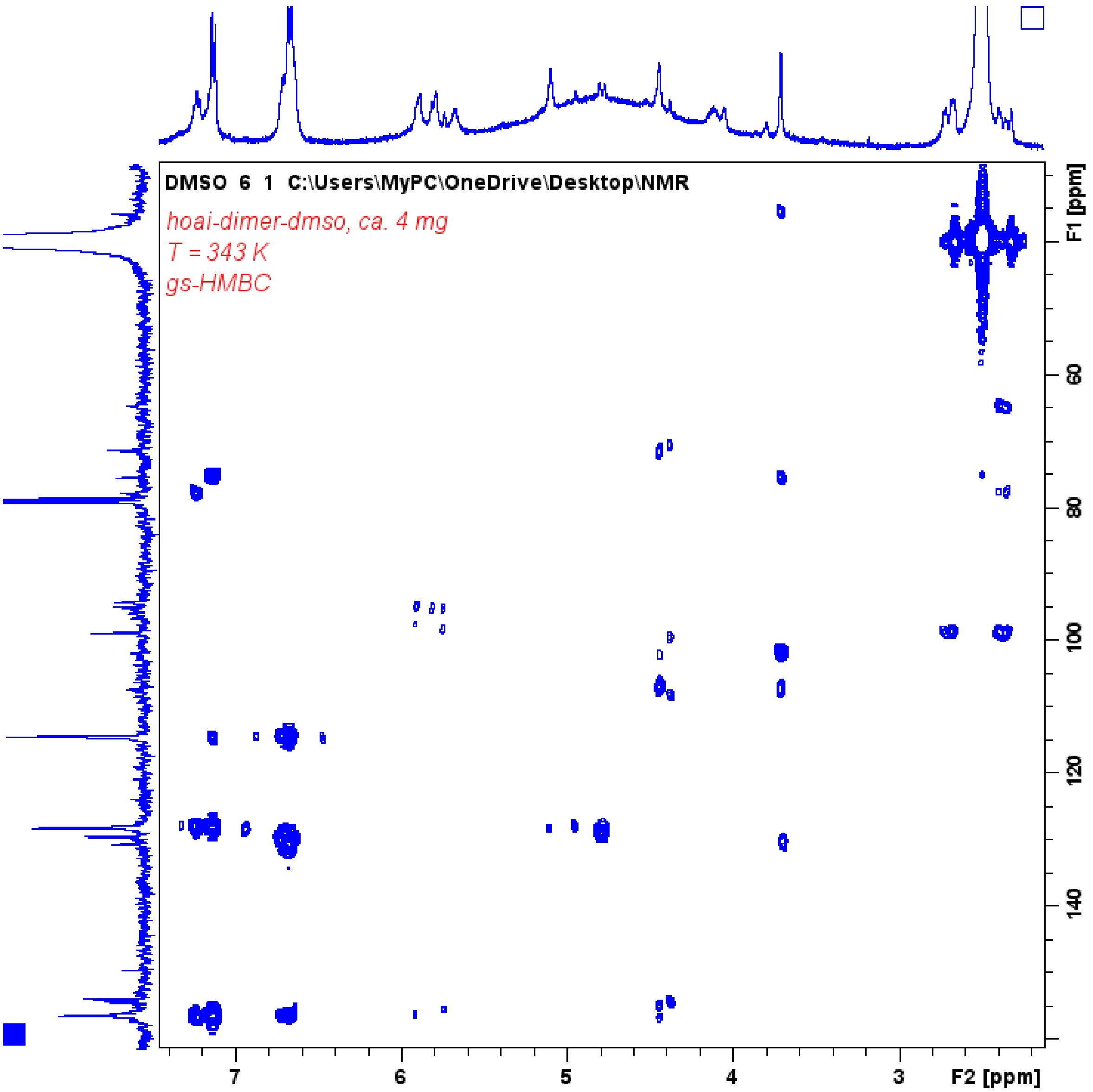

Figure S15. An expanded region of the ^{1}H-^{13}C HMBC NMR spectrum of epiafzelechin dimers

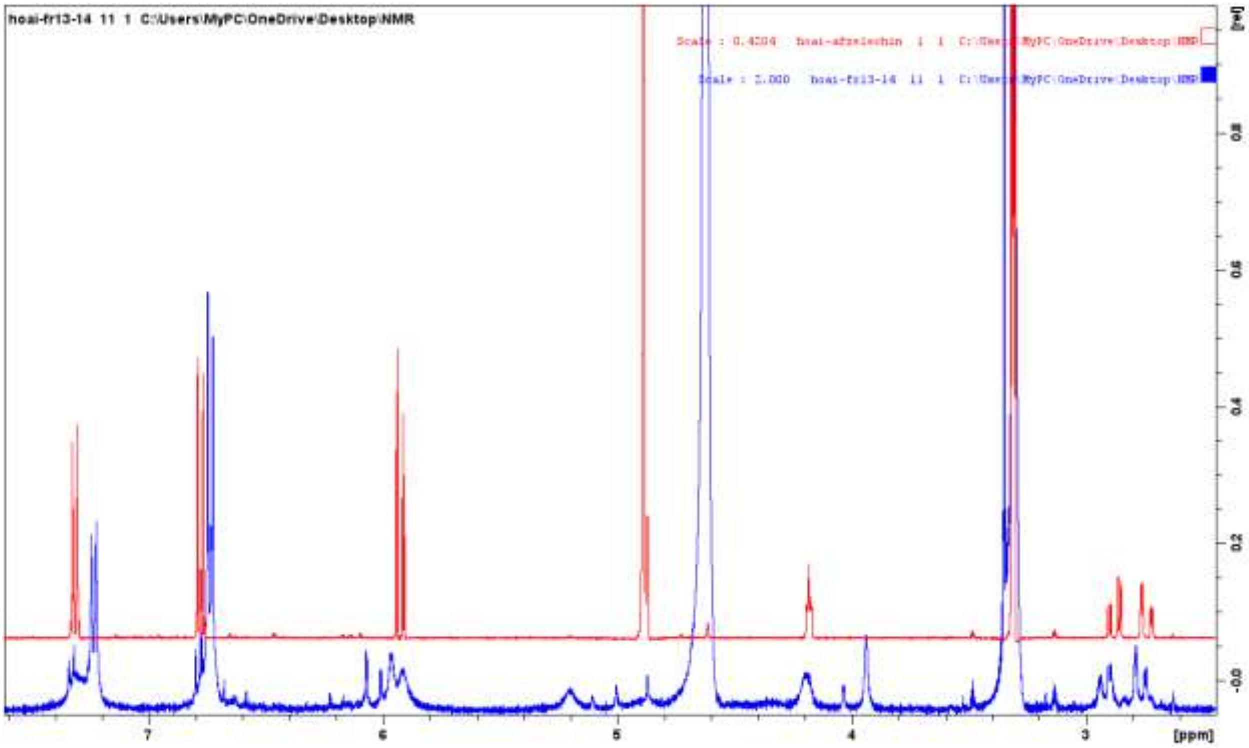

Figure S16. An expanded region of the ^{1}H NMR spectrum of epiafzelechin dimers (methanol-*d4*) (blue) and ^{1}H NMR spectrum of epiafzelechin (red)

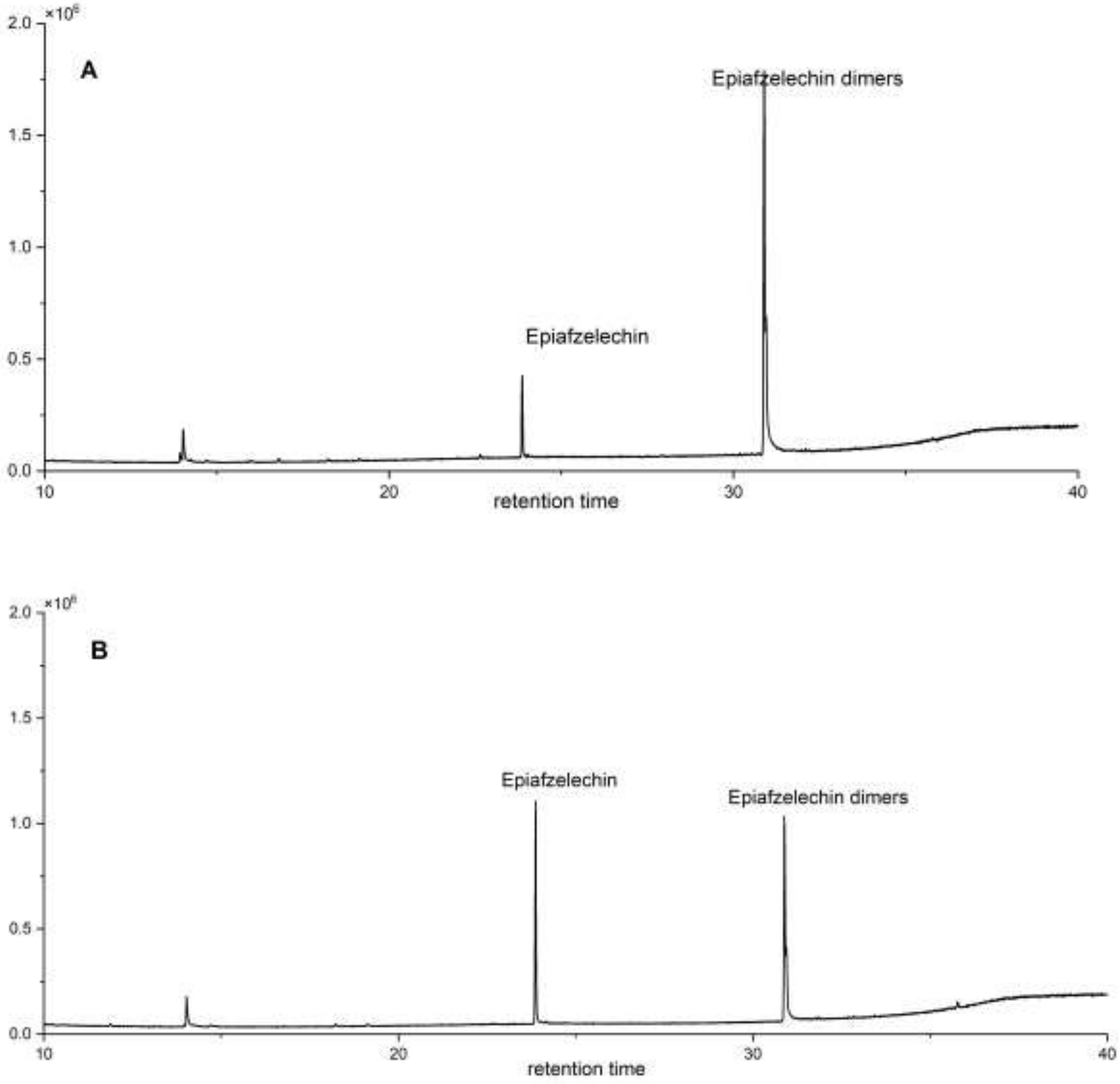

Figure S17: Gas chromatography of epiafzelechin dimers before (A) and after incubation in trifluoroacetic acid (B)

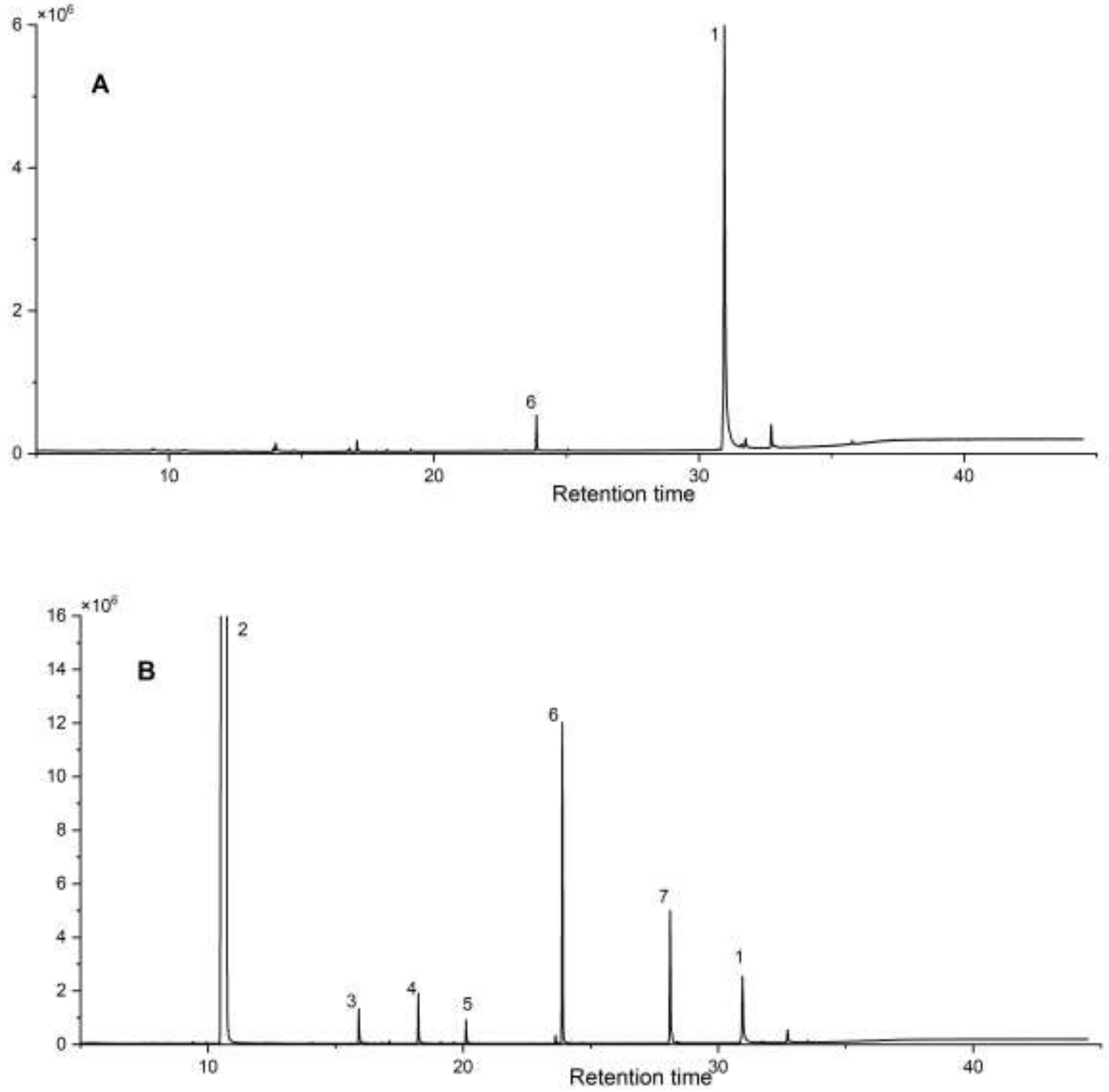

Figure S18: Gas chromatography of epiafzelechin dimers before (A) and after thiolysis (B)

(1) epiafzelechin dimer, (2) benzyl mercaptan, (3) benzyl sulfit (4) disulfite (5) 3-(Benzylthio)propene (6) epiafzelechin (7) epiafzelchin benzylthioether
